# Healthcare Practitioner and Other Professionals' Perspectives on Gabapentinoid Misuse and Dependence: A Systematic Review of Qualitative Studies

**DOI:** 10.1002/ejp.70116

**Published:** 2025-09-02

**Authors:** Amy G. McNeilage, Evan Browne, Suzanne Nielsen, Claire E. Ashton‐James, Bridin Murnion

**Affiliations:** ^1^ Sydney Medical School, Faculty of Medicine and Health The University of Sydney Sydney New South Wales Australia; ^2^ Monash Addiction Research Centre, Eastern Health Clinical School Monash University Frankston Victoria Australia; ^3^ School of Clinical Medicine, St Vincent's Healthcare Clinical Campus, Faculty of Medicine and Health University of New South Wales Sydney New South Wales Australia

**Keywords:** chronic pain, gabapentinoids, prescription drug misuse, qualitative, systematic review

## Abstract

**Background and Objective:**

The global rise in gabapentinoid prescriptions for chronic pain has been striking. However, this trend has been accompanied by growing concerns about misuse and dependence. This qualitative systematic review aimed to synthesise the perspectives of healthcare practitioners and other professionals on these emerging challenges.

**Databases and Data Treatment:**

Six databases (MEDLINE, Scopus, Web of Science, CINAHL, EMBASE, PsycINFO) were searched to May 2025. Eligible studies used qualitative or mixed methods to explore professional views on gabapentinoid misuse or dependence. Studies focusing solely on patient perspectives or therapeutic use were excluded. Methodological quality was assessed using the Critical Appraisal Skills Programme checklist. Data were analysed using thematic synthesis, and confidence in the findings was evaluated using GRADE‐CERQual.

**Results:**

After screening 1584 records, 19 original studies were included. Most were conducted in substance use treatment and law enforcement settings, where professionals frequently encounter vulnerable populations and more severe patterns of misuse than in general clinical care. Reported drivers of misuse included polydrug use, opioid substitution, psychological distress and poor access to non‐pharmacological care. Signs of misuse included early refills, dose escalation and reluctance to taper. Professionals described ethical tensions in prescribing and dispensing and proposed harm reduction strategies, including prescriber education, regulatory reform, expanded treatment access and public awareness.

**Conclusions:**

Gabapentinoid misuse is shaped by clinical, social and structural factors. Tackling these challenges requires systemic responses that go beyond individual prescribers. Lessons from opioid policy responses should inform balanced, compassionate and evidence‐informed strategies to reduce harm and improve care.

**Significance Statement:**

This is the first systematic review to synthesise professional perspectives on gabapentinoid misuse and dependence, drawing on studies from Europe, North America, the Middle East and Africa. Integrating insights from healthcare, law enforcement and policy settings, it reveals the structural drivers behind rising misuse—including opioid regulation, socioeconomic hardship and limited access to alternatives—and highlights gaps in clinical guidance. The findings offer actionable, cross‐sector strategies to support safer prescribing, informed policy and more effective harm reduction.

## 1 Introduction

The gabapentinoid drugs—gabapentin and pregabalin—are widely prescribed globally (Chan et al. 2023). A study of pharmaceutical sales data from 85 countries found on average gabapentinoid consumption increased 17.2% annually between 2008 and 2018 (Chan et al. 2023). Both drugs are indicated in most countries for the management of seizures and neuropathic pain (Chan et al. 2023). In addition, pregabalin is approved in several countries for the treatment of fibromyalgia and generalised anxiety disorder (Chan et al. 2023). There is evidence supporting their efficacy in some neuropathic pain conditions (e.g., postherpetic neuralgia, painful diabetic neuralgia), though not others (e.g., sciatica; Derry et al. 2019; Wiffen et al. 2017). Updated guidelines from the Neuropathic Pain Special Interest Group continue to recommend gabapentinoids as a first‐line treatment for neuropathic pain, while cautioning against harms, particularly when co‐prescribed with opioids due to increased risk of drug‐related deaths (Soliman et al. 2025).

Gabapentinoids are also frequently prescribed off‐label for non‐neuropathic pain conditions and, in some cases, for managing drug and alcohol withdrawal (Goodman and Brett 2019). In most cases, the evidence to support off‐label use is either lacking or unsupportive (Mack 2003; Peckham et al. 2018). The opioid epidemic has been identified as a possible driver of the overprescription of gabapentinoids, with prescribers under pressure to find safer alternatives for their patients with chronic pain (Goodman and Brett 2017). However, there is a growing body of research indicating gabapentinoids are not as safe as originally considered (Evoy et al. 2021), and concerns about the potential for misuse and dependence are rising (Murnion et al. 2022).

Both drugs are increasingly involved in accidental and intentional overdose deaths (Daly et al. 2018; Evoy et al. 2021). Increases in ambulance attendances and hospitalisations involving gabapentinoids have also been observed in a number of countries (Evoy et al. 2021), and are correlated with prescribing rates (Crossin et al. 2019). Euphoria at high doses has been documented and identified as a possible motive for misuse (Bonnet and Scherbaum 2017; Schjerning et al. 2016), and physical dependence and withdrawal symptoms have been widely reported (Evoy et al. 2021). High‐dose opioid use and opioid use disorder are known risk factors for gabapentinoid misuse and dependence (Evoy et al. 2021; Hofmann and Besson 2021), and concurrent gabapentinoid and opioid prescribing is common (Chen et al. 2022; Montastruc et al. 2018; Rahman et al. 2021; Schaffer et al. 2022), particularly in poorer communities (Schaffer et al. 2021; Torrance et al. 2020). This is concerning as the risk of fatal overdose, most likely from respiratory depression, is substantially higher when gabapentinoids are co‐ingested with opioids (Gomes et al. 2018, 2017; Hahn et al. 2022; Zhou et al. 2021).

There is clearly an urgent need to better understand the factors driving gabapentinoid misuse and dependence in order to develop effective harm reduction strategies and improve clinical practice. The aim of this systematic review was to synthesise the qualitative literature exploring the perspective of healthcare practitioners (HCPs) and others working at the coal face of gabapentinoid misuse and dependence. These professionals are uniquely placed to shed light on the current landscape, as well as provide suggestions for acceptable and feasible ways to mitigate emerging harms.

## 2 Methods

This systematic review is reported in line with the Preferred Reporting Items for Systematic Reviews and Meta‐Analyses (PRISMA) and Enhancing Transparency in Reporting the Synthesis of Qualitative Research (ENTREQ) guidelines (Page et al. 2021; Tong et al. 2012). Completed PRISMA and ENTREQ checklists are provided in the Tables S1 and S2. The protocol for this systematic review was registered on PROSPERO (CRD42023467535). Ethical approval was not required for this review as no human participants were involved, and no primary data was collected.

### 2.1 Eligibility Criteria

The eligibility criteria were formulated according to the PICo (Population, Phenomena of Interest, Context) framework used in reviews of qualitative evidence (Munn et al. 2018). Studies were eligible if they:

- Included HCPs or other professional groups (e.g., policymakers, regulators, first responders, community leaders, academics);
- Explored the phenomena of gabapentinoid misuse and/or dependence;
- Were conducted in any clinical or non‐clinical setting; and
- Used qualitative methods or mixed methods with a qualitative component.

Gabapentinoid misuse was defined as use without a prescription, in a route or dose other than prescribed, or for non‐therapeutic reasons (Dunn et al. 2017). Gabapentinoid dependence was defined according to the International Classification of Diseases 11th Revision (ICD‐11) criteria for substance dependence and the Diagnostic and Statistical Manual of Mental Disorders Fifth Edition (DSM‐5) criteria for substance use disorder (Saunders 2017). Data related to any of the aforementioned ICD‐11 or DSM‐5 criteria were included. Studies did not need to explicitly define or label the behaviour as misuse or dependence to be included. Rather, we included studies if the data described behaviours or experiences consistent with our operational definitions.

We excluded studies that focused solely on patient perspectives, as these have been synthesised in a separate review (McNeilage, Nielsen, et al. 2023; McNeilage et al. 2024), and studies addressing only therapeutic use without reference to misuse or dependence.

### 2.2 Search Strategy

The search strategy was pre‐planned and designed to be comprehensive. It included keywords and controlled vocabulary related to the following concepts: qualitative research, gabapentinoids, misuse and dependence. The search strategy for each database is included in Tables S3–S8. The six databases searched were MEDLINE, Scopus, Web of Science, CINAHL, EMBASE, and PsycINFO. All databases were searched from inception to September 25, 2023, and later updated on May 12, 2025. ProQuest Dissertations and Theses and Google Scholar were searched on May 12, 2025, to identify grey literature. For ProQuest, the same search strategy used in the main databases was applied (see Table S9). Google Scholar was searched in incognito mode using a simplified version of the strategy, adapted to meet character limits and supported operators (see Table S10). One reviewer (AM) screened the first 200 Google Scholar results and extracted any relevant studies not identified in other searches. The reference lists of all included studies were also screened (AM).

We placed no geographical or language restrictions on our search strategy. All titles and abstracts were screened regardless of language. For non‐English studies, we used Google Translate for initial screening and, if a study appeared potentially eligible, we reviewed the full‐text using a combination of automated translation tools and input from colleagues fluent in the relevant language. No non‐English studies ultimately met the inclusion criteria, so professional translation was not required (Note: This approach differed from our original PROSPERO registration, which had indicated exclusion of non‐English studies. See Section 2.9 for further explanation).

### 2.3 Screening Process

All records were uploaded to Covidence, and duplicates were automatically removed by the software. Two of four authors (AM, EB, SN, BM) independently screened each title and abstract with an interim review to ensure consistency in the application of the eligibility criteria. Conflicts were resolved through discussion (AM, EB). Two authors (AM, EB) then independently performed full‐text reviews with conflicts resolved in consultation with a third author (BM).

### 2.4 Data Extraction

Data were extracted by one author (AM or EB) and verified and refined by a second (AM or EB). Study characteristics were extracted and summarised in an Excel spreadsheet. Qualitative findings were extracted verbatim into Word files. In instances where two separate records related to the same study (e.g., a conference abstract and a journal article), they were combined during data extraction with the most comprehensive record identified as the primary reference for the study.

### 2.5 Quality Assessment

The methodological quality of included studies was assessed using the modified 11‐item version of the Critical Appraisal Skills Programme (CASP) qualitative checklist (Long et al. 2020). Based on responses to the 11 items, the overall quality of each study was rated as either low, medium, or high. The assessment focused specifically on the qualitative components of each study and does not necessarily reflect the overall quality of mixed methods studies. The assessment was first conducted independently by two authors (AM and EB), and consensus was reached through discussion. Decisions were recorded in Covidence. Higher quality studies were prioritised in the thematic synthesis, while poorer quality studies were included but with less emphasis (see Section 2.6 for more information).

### 2.6 Data Synthesis

NVivo 14 software was used to manage the qualitative data during analysis. Data were analysed using thematic synthesis (Thomas and Harden 2008). This approach was chosen as the aim of the review was to provide practical insights that would be valuable to HCPs and policymakers rather than to generate theory. Moreover, thematic synthesis is well‐suited to addressing broad and exploratory review questions (Booth et al. 2016).

Two authors (AM and EB) independently coded the entire dataset inductively. They then met to compare and discuss their interpretations, reflecting on their respective disciplinary biases. Through discussion, they reached a shared understanding and finalised the coding framework. In collaboration with a third author (BM), the codes were then iteratively grouped to generate descriptive themes. A pragmatic decision was made to maintain descriptive themes rather than construct more analytic themes so that the findings would be easy to interpret and apply to real‐world settings (Flemming and Noyes 2021; Sandelowski 2000, 2010).

No themes were generated purely on the basis of data drawn from studies appraised as low quality. Additionally, data from high‐quality studies were given greater emphasis in the reporting of findings (Long et al. 2020). Where possible, contradictory data or outliers were reported. Exemplar quotes from included studies were used to enrich the reporting of themes. All quotes were from participants of included studies and not authors, and some were superficially edited to improve readability.

### 2.7 Confidence in the Evidence

Following thematic synthesis, the GRADE‐CERQual approach was used to assess confidence in the overall findings of the review (Lewin et al. 2018). The assessment takes into consideration the methodological limitations of included studies, the level of congruence between the included data and the synthesised findings, the adequacy of the included data in terms of both quantity and richness and the relevance of the included data to the specific review question. The assessment was conducted collaboratively by two authors (AM and EB) using the online iSoQ tool (https://isoq.epistemonikos.org/).

### 2.8 Reflexivity Statement

Our review team consisted of four white women (AM, SN, CAJ, BM) and one white man (EB), all of whom are university‐educated and based in metropolitan Australia. None had a lived experience of gabapentinoid use. The interdisciplinary team included three HCPs: an early career doctor in clinical pharmacology and toxicology with experience in acute, inpatient clinical care (EB); a registered pharmacist with experience in the treatment of substance use disorders in community pharmacy and specialist drug treatment settings (SN); and a medical specialist in clinical pharmacology, pain medicine and addiction medicine (BM). Both SN and BM have also held expert roles on government bodies advising on drug dependence and scheduled medicines. The team also included two social scientists (AM, CAJ) with research experience and training in social psychology, pain management and qualitative methods. None of the review team members had experience working in law enforcement or in any of the other professions represented in the included studies (e.g., teaching, veterinary medicine, religious leadership).

### 2.9 Differences From Published Protocol

Our PROSPERO registration (CRD42023467535) indicated that non‐English language studies would be excluded. In practice, we adopted a more inclusive approach, as described in Section 2.2. Additionally, we expanded our grey literature search to include Google Scholar. No other deviations from the registered protocol were made.

## 3 Results

**FIGURE 1:**
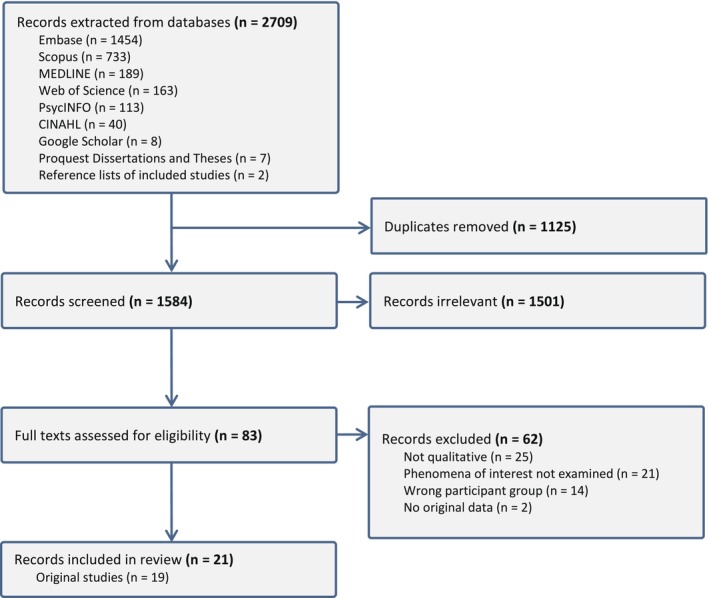
Preferred Reporting Items for Systematic Reviews and Meta‐Analyses (PRISMA) flow diagram.

The search strategy returned 1584 records after deduplication. Twenty‐one records representing 19 original studies met the full inclusion criteria. See Figure 1 for the PRISMA flow diagram.

### 3.1 Study Characteristics

The characteristics of individual studies are summarised in Table 1. Studies were published between 2015 and 2025. Fifteen used a qualitative design and four used a mixed methods design. The main data collection method was a semi‐structured interview (*n* = 13), followed by a mixed‐method survey (*n* = 5), published commentary (*n* = 1), focus group (*n* = 1) and participant observation (*n* = 1), with several studies using multiple methods. Eleven studies discussed both gabapentinoid misuse and dependence, seven discussed misuse only and one discussed dependence only. Six of the studies had research aims that were directly relevant to the review questions, while 13 had aims that were partially relevant. The UK and US accounted for the majority of studies (*n* = 13), with one study each from Canada, Iran, Somalia, Sri Lanka, Sudan, Europe and an international source.

**TABLE 1:** Characteristics of included studies.

Author(s), year	Publication type	Research design	Country	Setting	Sample characteristics	Research aims	Phenomena of interest	Data collection method	Data analysis method	Methodological quality (CASP appraisal)
Altayeb et al. (2025)	Journal article	Mixed methods	Sudan	Community pharmacies in the Omdurman locality of Khartoum state	172 community pharmacists	To assess community pharmacists' perceptions of pregabalin abuse and their recommendations to address this issue	Misuse and dependence	Self‐administered questionnaire with one open‐ended question about potential solutions to pregabalin abuse	Narrative responses were categorised into themes and summarised	Low
Buttram et al. (2017)	Journal article	Mixed methods	United States	Law enforcement and regulatory agencies from 13 states	21 prescription drug diversion investigators	To investigate drug diversion officers' opinions regarding abuse or diversion of gabapentin, including the characteristics of abusers, modes of diversion and street value of the drugs	Misuse	Questionnaire distributed via email in Apr 2016, including a single open‐ended item about abuse and diversion of gabapentin	Thematic analysis	Low
Buttram et al. (2019)	Journal article	Qualitative	United States	Public and private substance abuse treatment centres in South Florida	12 staff members currently working with opioid‐dependent clients, including therapists, a social worker, a nurse and managerial and operational staff	To examine substance abuse treatment providers' experiences of how gabapentin is used in these settings and the benefits and risks for clients	Misuse and dependence	Semi‐structured interviews conducted Aug 2018 to Jan 2019	Descriptive qualitative approach	High
Buttram et al. (2023)	Journal article	Qualitative	United States	Law enforcement and regulatory agencies from 25 states, including 5 states where gabapentin was classified as a controlled substance	46 prescription drug diversion investigators	To investigate prescription drug diversion investigator's experiences with gabapentin, including non‐medical use, diversion and state‐level controlled substances laws	Misuse and dependence	Open‐ended questionnaire distributed via email in Apr 2022	Descriptive qualitative approach	Medium
Chandrasiri et al. (2024)	Journal article	Qualitative	Sri Lanka	Urban and semiurban schools in the southern province of Sri Lanka	47 teachers involved in counselling activities	To explore illicit drug use behaviour among schoolchildren in the southern province of Sri Lanka	Misuse	Semi‐structured interviews	Thematic analysis	Low
Coombes and Cooper (2019)	Journal article	Qualitative	United Kingdom	Government and charity drug treatment services across England	15 staff working in drug treatment services including nurses, doctors, therapists, recovery workers and managers	To explore the views and experiences of health care professionals working in drug treatment services in relation to supporting clients with prescription and over‐the‐counter drug dependence	Misuse and dependence	Semi‐structured interviews conducted in 2018	Thematic analysis	High
Covvey et al. (2022); Covvey et al. (2023)	Conference abstract; Journal article	Qualitative	United States	Various healthcare settings across the US, including primary care, substance use treatment, pain management and psychiatry	43 healthcare and policy experts, including pharmacists, physicians, nurse practitioners and drug policy experts	To explore prescribers, pharmacists and drug policy experts awareness, opinions and experiences regarding gabapentinoid misuse	Misuse and dependence	Semi‐structured interviews conducted Feb to Apr 2021	Thematic analysis	High
De Kock et al. (2023)	Institutional report	Mixed Methods	Europe	Reception centres for asylum seekers across the European Union and Norway	16 professionals and volunteers working with international protection applicants in reception centres, including doctors, nurses, psychologists and reception officers	To document the experiences and needs of professionals working in reception centres regarding drug‐related issues among international protection applicants	Misuse	Four online focus groups with 3 to 6 participants	Not reported	Medium
Falzon et al. (2023)	Journal article	Qualitative	United Kingdom	Three Scottish cities where drug checking service implementation is being planned	27 professionals, including police, healthcare workers and not‐for‐profit staff	To explore perceptions of the potential harm reduction impacts of drug checking services in Scotland	Misuse	Semi‐structured interviews conducted Mar to Sep 2021	Thematic analysis	High
Ghinea et al. (2015)	Journal article	Qualitative	International	Published clinical debate	Clinicians publishing commentary around gabapentin prescribing	To explore the clinical reasoning underpinning the ongoing prescribing of gabapentin for neuropathic pain	Dependence	116 comments, editorials and letters from 61 journals published in English up to Aug 2013	Qualitative empirical analysis	Low
Gittins et al. (2024); Gittins (2024)	Conference abstract; Thesis	Qualitative	United Kingdom	Five community substance misuse services in England operated by a national non‐government treatment provider	20 front‐line staff working in community substance misuse services, including doctors, nurses and recovery workers	To explore the perspectives of staff working in substance misuse services on treating problematic over‐the‐counter and prescription‐only medication use	Misuse and dependence	Semi‐structured interviews conducted Oct 2020 to Jan 2021	Thematic analysis	High
Jeffries et al. (2025)	Journal article	Qualitative	United Kingdom	30 adult prison sites across England	25 prison healthcare staff involved in the delivery or implementation of a safe prescribing intervention, including general practitioners, nurses and pharmacists	To understand the implementation and impact of a suite of seven prescribing safety indicators, specifically developed for use in prison settings	Misuse and dependence	Semi‐structured interviews (*n* = 9) conducted May to Aug 2022 and an open‐ended survey (*n* = 18) conducted in Ju to Aug 2022	Thematic analysis and template analysis	High
Jorgenson et al. (2021)	Journal article	Qualitative	Canada	Any physician licensed in the Province of Saskatchewan	17 physicians, including 16 family physicians and one internal medicine expert	To collect opinions of Saskatchewan physicians regarding their personalised prescriber profile (an audit and feedback tool to reduce the sub‐optimal prescribing of high‐risk drugs)	Misuse	Semi‐structured interviews conducted Jan 2019	Thematic analysis	Medium
Lehnus et al. (2024)	Journal article	Mixed methods	United Kingdom	Charity, private or academic small, large or mixed animal veterinary practices across the United Kingdom	361 veterinarians, including general practitioners and specialists	To investigate veterinarians' experience and perception of the risk of veterinary prescription medication misuse and abuse by the public and veterinary professionals	Misuse and dependence	Anonymous online survey including open‐ended questions	Thematic analysis	Medium
May et al. (2022)	Journal article	Qualitative	United Kingdom	A drug charity with treatment services located across England and Scotland	17 drug service providers, including clinical, management and frontline staff	To explore the impact of the COVID‐19 pandemic on the health and well‐being of people who use drugs	Misuse	Semi‐structured interviews conducted May to Sep 2021	Reflexive thematic analysis	High
Mikhael et al. (2024)	Journal article	Qualitative	Iraq	Community pharmacies across Baghdad	21 community pharmacists with at least 1 year of working experience	To understand the perception, experience and practice of Iraqi community pharmacists toward customers with substance use disorders	Misuse and dependence	Semi‐structured interviews conducted Jul to Sep 2023	Thematic analysis	High
Mohamed and Bashir (2024)	Journal article	Qualitative	Somalia	Community stakeholders actively addressing substance abuse in Mogadishu	5 stakeholders including a police officer, a regulator, a religious leader and two social workers	To explore the roles, challenges and collaborative efforts of key stakeholders engaged in addressing substance abuse in Mogadishu	Misuse and dependence	Semi‐structured interviews	Thematic analysis	Low
Parbery‐Clark et al. (2025)	Journal article	Qualitative	United Kingdom	5 general practices serving areas of substantial socioeconomic disadvantage in the North East of England	13 primary healthcare professionals including doctors, pharmacists and allied health professionals	To explore primary healthcare professional perspectives on designing and delivering an intervention to reduce high‐risk opioid and gabapentinoid prescribing in disadvantaged settings	Misuse and dependence	Participant observation and semi‐structured interviews	Thematic analysis	High
Pivovarova et al. (2023)	Journal article	Qualitative	United States	7 drug courts in one northeastern state	21 drug court staff, including judges, probation officers and clinicians	To examine drug court staff perspectives on barriers and facilitators to enhancing collaboration with providers of medications for opioid use disorder	Misuse	Semi‐structured interviews conducted Mar to Jun 2021	Grounded theory	High

### 3.2 Study Quality

Following quality appraisal, 10 studies were considered to be high quality, 4 were considered to be medium quality and 5 were considered to be low quality (See Table 1). The most common methodological issue was a lack of reflexivity, followed by unclear theoretical underpinnings. The full quality appraisal is available in the Table S11.

### 3.3 Characteristics of Participants

Included studies involved a wide range of professional participants (*N* = 899) with experience relevant to gabapentinoid misuse and dependence. Healthcare practitioners featured prominently, including medical specialists, pharmacists, nurses, psychologists and other allied health professionals. Several studies involved staff working in substance use treatment services, such as recovery workers, therapists, social workers and service managers. Law enforcement and regulatory personnel were also represented, including prescription drug diversion investigators, police officers and drug court staff such as judges and probation officers. In addition, some studies included drug policy experts, public health professionals and staff from not‐for‐profit organisations involved in harm reduction or community support. Other community‐based professionals, such as teachers engaged in school counselling and staff working with asylum seekers in reception centres, contributed perspectives on misuse and dependence in specific populations. Finally, one study included veterinarians, providing insight into concerns around veterinary prescription medication misuse.

### 3.4 Thematic Synthesis

Five main descriptive themes and five sub‐themes were constructed to capture the perspectives of professionals on gabapentinoid misuse and dependence. The themes reflect variability in awareness and perceptions of risk, insights into the drivers and signs of misuse and dependence, ethical tensions in prescribing and dispensing and strategies for prevention and harm reduction.

#### 3.4.1 Confidence in the Evidence

The GRADE‐CERQual appraisal was conducted on the nine main review findings. Two findings were assigned a high confidence rating, five were assigned a moderate confidence rating, and two were assigned a low confidence rating (See Table 2). High‐confidence findings were derived from methodologically robust studies, rich data, a coherent synthesis and strong relevance to the review question. Moderate‐confidence ratings reflected minor to moderate concerns in one or more domains—most commonly related to the depth of supporting data, the specificity of participant insights (i.e., whether they were directly focused on gabapentinoids), or geographic transferability. Low‐confidence ratings were assigned where concerns about methodological rigour or data adequacy were more substantial. The full GRADE‐CERQual appraisal is available in the Table S12.

**TABLE 2:** GRADE‐CERQual summary of qualitative findings table.

#	Summarised review finding	GRADE‐CERQual assessment of confidence	Explanation of GRADE‐CERQual assessment	References
1	*Variability in awareness* (Section 3.4.2): Awareness of gabapentinoid misuse and dependence varied across countries, professional roles and settings. While some professionals gained awareness through education or media, most cited first‐hand exposure. Healthcare practitioners in psychiatry, community pharmacy and substance use treatment were generally more alert to misuse than those in primary care. Misuse was also observed among vulnerable groups, including schoolchildren and asylum seekers	Moderate confidence	Minor concerns regarding methodological limitations, Minor concerns regarding coherence, No/Very minor concerns regarding adequacy and Minor concerns regarding relevance	Altayeb et al. (2025); Buttram et al. (2017); Buttram et al. (2019); Buttram et al. (2023); Chandrasiri et al. (2024); Coombes and Cooper (2019); Covvey et al. (2023); De Kock et al. (2023); Falzon et al. (2023); Ghinea et al. (2015); Gittins (2024); Jeffries et al. (2025); Jorgenson et al. (2021); Lehnus et al. (2024); May et al. (2022); Mikhael et al. (2024); Mohamed and Bashir (2024); Parbery‐Clark et al. (2025); Pivovarova et al. (2023)
2	*Divergent risk perceptions* (Section 3.4.2.1): Healthcare practitioners generally perceived gabapentinoids as lower risk than opioids, often viewing them as a safer alternative in pain management. Law enforcement officers and professionals in disadvantaged or high‐risk settings expressed more concern about potential harms associated with gabapentinoids, particularly when used in combination with opioids	Moderate confidence	Minor concerns regarding methodological limitations, No/Very minor concerns regarding coherence, Minor concerns regarding adequacy and Moderate concerns regarding relevance	Buttram et al. (2017); Buttram et al. (2019); Covvey et al. (2023); Falzon et al. (2023); Ghinea et al. (2015); Jeffries et al. (2025); Lehnus et al. (2024); Parbery‐Clark et al. (2025); Pivovarova et al. (2023)
3	*Perceived drivers* (Section 3.4.3): Gabapentinoid misuse was attributed to multiple drivers, including the pursuit of euphoria, enhancement of opioid effects, self‐medication, drug availability and undetected use in drug screens. Systemic issues such as poor access to non‐pharmacological care, opioid tapering practices and socioeconomic disadvantage were also implicated as key contributors	High confidence	Minor concerns regarding methodological limitations, No/Very minor concerns regarding coherence, No/Very minor concerns regarding adequacy and Minor concerns regarding relevance	Altayeb et al. (2025); Buttram et al. (2017); Buttram et al. (2019); Buttram et al. (2023); Coombes and Cooper (2019); Covvey et al. (2023); De Kock et al. (2023); Gittins (2024); Lehnus et al. (2024); May et al. (2022); Mikhael et al. (2024); Mohamed and Bashir (2024); Parbery‐Clark et al. (2025)
4	*Potential signs* (Section 3.4.4): Participants identified behavioural and prescribing patterns as signs of misuse or dependence, including early refills, unsanctioned dose escalation, multiple prescribers and signs of intoxication or withdrawal. Due to stigma and legal concerns, patients often conceal misuse, prompting practitioners to rely on indirect indicators	Moderate confidence	Minor concerns regarding methodological limitations, Minor concerns regarding coherence, Moderate concerns regarding adequacy and Minor concerns regarding relevance	Buttram et al. (2017); Buttram et al. (2019); Covvey et al. (2023); Jorgenson et al. (2021); Lehnus et al. (2024); Mikhael et al. (2024); Parbery‐Clark et al. (2025)
5	*Navigating ethical dilemmas* (Section 3.4.5): Professionals described complex ethical tensions in managing gabapentinoid prescribing and dispensing, particularly in the context of chronic pain, substance use and social disadvantage. They often faced a difficult balance between alleviating patient suffering and avoiding the potential for misuse or dependence. Decision‐making was further complicated by limited clinical guidance, poor access to alternatives, concerns about damaging therapeutic relationships and, in some cases, patient threats of self‐harm	High confidence	No/Very minor concerns regarding methodological limitations, Minor concerns regarding coherence, No/Very minor concerns regarding adequacy and No/Very minor concerns regarding relevance	Buttram et al. (2019); Covvey et al. (2023); Ghinea et al. (2015); Mikhael et al. (2024); Parbery‐Clark et al. (2025)
6	*Improving clinical decision‐making* (Section 3.4.6.1): Strategies to support safer prescribing included improved education, clearer guidelines, better monitoring systems (e.g., PDMPs, decision support tools) and enhanced interdisciplinary collaboration. Early prescribing decisions were seen as crucial, particularly for high‐risk patients, and professionals called for more robust tools and training to manage tapering and dependence	Moderate confidence	No/Very minor concerns regarding methodological limitations, Minor concerns regarding coherence, Minor concerns regarding adequacy and No/Very minor concerns regarding relevance	Altayeb et al. (2025); Buttram et al. (2019); Coombes and Cooper (2019); Covvey et al. (2023); Gittins (2024); Jeffries et al. (2025); Jorgenson et al. (2021); Mikhael et al. (2024); Parbery‐Clark et al. (2025)
7	*Strengthening regulation and enforcement* (Section 3.4.6.2): Regulatory and enforcement responses were seen as important but potentially double‐edged. While scheduling gabapentinoids was supported by law enforcement, healthcare practitioners and drug policy experts expressed concern about patient access and unintended consequences. Participants stressed the need for balanced regulation that protects public health without being overly punitive	Low confidence	Moderate concerns regarding methodological limitations, Minor concerns regarding coherence, Moderate concerns regarding adequacy and Minor concerns regarding relevance	Altayeb et al. (2025); Buttram et al. (2023); Covvey et al. (2023); Jorgenson et al. (2021); Mikhael et al. (2024); Mohamed and Bashir (2024)
8	*Expanding access to care* (Section 3.4.6.3): Improving access to non‐pharmacological pain care, substance use treatment and mental health support was seen as vital for prevention and harm reduction. Barriers included cost, availability, stigma and systemic underinvestment. Participants emphasised the need for holistic, inclusive services tailored to the needs of people with chronic pain or prescription drug dependence	Moderate confidence	Minor concerns regarding methodological limitations, Minor concerns regarding coherence, Minor concerns regarding adequacy and No/Very minor concerns regarding relevance	Altayeb et al. (2025); Coombes and Cooper (2019); Covvey et al. (2023); Gittins (2024); Mohamed and Bashir (2024); Parbery‐Clark et al. (2025)
9	*Raising awareness* (Section 3.4.6.4): Participants highlighted the importance of coordinated awareness initiatives to improve patient understanding and reduce stigma. Strategies included patient education at the point of prescribing, public health campaigns and community engagement. However, limited resources, unclear leadership and clinical constraints hindered widespread implementation	Low confidence	Minor concerns regarding methodological limitations, Moderate concerns regarding coherence, Minor concerns regarding adequacy and Moderate concerns regarding relevance	Altayeb et al. (2025); Coombes and Cooper (2019); Falzon et al. (2023); Gittins (2024); Mohamed and Bashir (2024); Parbery‐Clark et al. (2025)

#### 3.4.2 Variability in Awareness of Misuse and Dependence

All included studies addressed awareness of gabapentinoid misuse and dependence, spanning diverse professional roles and international settings. Some participants gained general awareness through education, media, or peer discussions (Buttram et al. 2017, 2019, 2023; Covvey et al. 2023), while many others cited direct clinical or professional experience (Altayeb et al. 2025; Buttram et al. 2017, 2019, 2023; Chandrasiri et al. 2024; Coombes and Cooper 2019; Covvey et al. 2023; De Kock et al. 2023; Gittins 2024; Mikhael et al. 2024).

In medical contexts, awareness often depended on clinical role and setting. Those working in psychiatry, community pharmacy and substance use treatment tended to show greater recognition of misuse than prescribers in primary care (Coombes and Cooper 2019; Covvey et al. 2023; Gittins 2024). One US addiction specialist expressed frustration that colleagues in primary care would “just pump this stuff out like it is candy” without acknowledging the risks (Covvey et al. 2023).

Studies from the US and UK found high levels of awareness among clinical and non‐clinical staff in substance use treatment settings (Buttram et al. 2019; Coombes and Cooper 2019; Gittins 2024; May et al. 2022). Participants reported growing misuse—usually involving oral consumption, dependence and polydrug use. One observed, “more recently we have seen dependence on other drugs like pregabalin, which are causing lots of issues really*”* (Coombes and Cooper 2019); another noted that “pregabalin and gabapentin… seem to be on the rise” (Gittins 2024).

In contrast, awareness in primary care was more limited or varied. In the US, many HCPs in primary care were unaware or only recently aware of gabapentin diversion (Covvey et al. 2023), though Canadian HCPs identified gabapentin as commonly misused (Jorgenson et al. 2021). Awareness extended beyond traditional settings: Scottish HCPs cited frequent online purchasing of gabapentinoids (Falzon et al. 2023), and UK veterinarians reported pet‐owner misuse risks (Lehnus et al. 2024).

Outside high‐income countries, community pharmacists in Sudan and Iraq identified gabapentinoids as highly requested and dependence‐prone (Mikhael et al. 2024; Altayeb et al. 2025). One Iraqi pharmacist noted, “The most commonly requested drugs for use among people with substance use disorder are Lyrica and Pulmocodin syrup” (Mikhael et al. 2024). In Somalia, pregabalin misuse was described as a “*massive*” and growing issue (Mohamed and Bashir 2024).

Misuse was also observed among vulnerable populations. Pregabalin was identified as one of the most frequently used substances among Sri Lankan schoolchildren (Chandrasiri et al. 2024), and professionals working with asylum seekers in Europe reported rising concerns, often tied to previous use in the country of origin or during transit (De Kock et al. 2023).

Awareness also varied among US drug diversion investigators (Buttram et al. 2017, 2023). Some described gabapentin as a highly misused and diverted substance, while others had no direct exposure, reflecting regional differences in substance use trends and drug laws. Investigators often learned of misuse through investigations or conversations with HCPs.

These findings highlight the uneven awareness of gabapentinoid misuse and dependence across professional roles, care settings and national contexts.

##### 3.4.2.1 Divergent Risk Perceptions

Healthcare practitioners generally perceived gabapentinoids as relatively low risk for misuse and dependence, especially compared to opioids. For instance, an analysis of published clinical debate found gabapentin was often not believed to be an addictive drug and was widely used due to its “favourable safety profile” (Ghinea et al. 2015). Similarly, substance use treatment providers in the US viewed gabapentin as a non‐narcotic, and thus less prone to misuse (Buttram et al. 2019). Healthcare practitioners in the US expressed relatively little concern, partly because overdose deaths involving gabapentinoids were seen as rare: “I am not fearful that someone is going to fatally overdose on gabapentin or Lyrica” (Covvey et al. 2023). On the contrary, gabapentinoids were often viewed as a pragmatic harm reduction strategy—safer than opioids both in terms of dependence and toxicity risks, and less scrutinised from a prescribing standpoint (Buttram et al. 2019; Covvey et al. 2023; Ghinea et al. 2015). As one HCP put it, “I think they feel that's the lesser of the caustic medication that they may choose next up the pain ladder” (Covvey et al. 2023).

However, this low‐risk perception was not universal. In the UK, HCPs working in disadvantaged communities flagged patients on high doses or with substance use histories as being particularly vulnerable to dependence‐related harms (Parbery‐Clark et al. 2025). In UK prisons, gabapentinoids were recognised as potentially risky when co‐prescribed with opioids (Jeffries et al. 2025). This view was echoed in US drug courts, where staff raised concerns about sedative effects and misuse when gabapentin was prescribed alongside medications for opioid use disorder (Pivovarova et al. 2023).

Law enforcement officers, on the other hand, appeared to perceive greater risk associated with gabapentinoid misuse and dependence. When describing the potential harms, drug diversion investigators highlighted overdose deaths and suicide attempts involving gabapentin, often in combination with other substances such as heroin (Buttram et al. 2017, 2023).

Perceptions of risk varied not only by profession but also by setting, reflecting differing clinical priorities, exposure to harms and assumptions about safety.

#### 3.4.3 Perceived Drivers of Misuse and Dependence

Professionals described a wide range of factors driving gabapentinoid misuse and dependence, from individual motivations to systemic and structural issues. At the individual level, motivations included altering mood or consciousness, enhancing or substituting other drugs (especially opioids) and self‐medicating for pain or psychological distress. Desired effects were said to include euphoria and stimulation as well as sedation and relaxation (Buttram et al. 2019, 2023; Mohamed and Bashir 2024). According to substance use treatment providers in the US, gabapentin was known to produce “a high of sorts”, although “not to the extent or magnitude of heroin or crack” (Buttram et al. 2019). This “high” was said to include “*drowsy*”, “*speedy*”, “*loopy*” and “*floating*” feelings (Buttram et al. 2019). One social worker reported it could produce “a drunk feeling… an up feeling where they have more energy and they can do more with their day” (Buttram et al. 2019), while a drug diversion investigator reported people who use drugs “often tell detectives that gabapentin gives them a drunk‐like high” (Buttram et al. 2023).

Both HCPs and law enforcement officers agreed that gabapentin misuse was strongly tied to prescription and illicit opioid use (Buttram et al. 2019, 2023; May et al. 2022). Law enforcement officers reported that gabapentin misuse predominantly occurred in the context of poly‐drug use, noting that “[it's] the same people that are over‐dosing on heroin or fentanyl” (Buttram et al. 2023). They explained that the drug was used both to increase the intensity or duration of opioid effects and to manage the symptoms of withdrawal from opioids (Buttram et al. 2017, 2023). For example, one investigator stated that “abusers are using gabapentin to enhance their high and assist with being dope sick”, while another noted that “most of the people we get with gabapentin are opiate abusers looking for a better euphoria” (Buttram et al. 2023). In another law enforcement study (Buttram et al. 2017), it was reported that “heroin users are mixing a combination of gabapentin and heroin and injecting the compound”. It was also reported that gabapentin was used in combination with buprenorphine/naloxone because “the blocker doesn't affect the gabapentin” (Buttram et al. 2023). Concurrent use with methamphetamine was also reported, though the reasons for this combination were not clearly explained (Buttram et al. 2023).

Gabapentinoid misuse was also attributed to the drugs' widespread availability, low cost and reduced likelihood of detection in drug screens, especially compared to opioids (Buttram et al. 2017, 2019, 2023; De Kock et al. 2023; Gittins 2024; Mikhael et al. 2024; Mohamed and Bashir 2024; May et al. 2022). For example, an Iraqi pharmacist explained, “The main reason for requesting Lyrica is its availability in most pharmacies, the low cost of its generic products and its minimal side effects” (Mikhael et al. 2024). Pregabalin misuse reportedly increased in the UK during the COVID‐19 pandemic, driven by shortages of drugs like heroin (May et al. 2022). Indeed, substance use treatment providers noted that misuse often occurred when preferred substances were unavailable. For example, “One thing that is quite common is you know… ‘I couldn't get any heroin, but got gabapentin’” (Gittins 2024). Similar patterns were observed by law enforcement in the US when opioids like hydrocodone or heroin were unavailable (Buttram et al. 2023).

Self‐medication was identified as another key driver of gabapentinoid misuse, particularly in response to psychological distress, chronic pain, or withdrawal from other substances (Buttram et al. 2019; Coombes and Cooper 2019; Gittins 2024; Lehnus et al. 2024). A UK veterinarian reflected, “Human medicine does not cater well for those in chronic pain, and I fear some [pet] owners that suffer would use their animal's medications because they are desperate, not because they are a stereotypical drug addict” (Lehnus et al. 2024). In another study, a medical specialist working in primary care believed some patients misused gabapentinoids to manage pain after a forced opioid taper (Covvey et al. 2023).

The influence of broader healthcare and socioeconomic inequities was also acknowledged. Fractured healthcare—marked by short consultations, poor access to non‐pharmacological treatments and insurance constraints—often left prescribers reliant on low‐cost drug options like gabapentinoids (Covvey et al. 2023; Coombes and Cooper 2019). These issues, combined with prescriber caution around opioids and patient expectations of pharmacological treatment for pain, were seen to fuel excessive or off‐label prescribing (Covvey et al. 2023; Coombes and Cooper 2019). Such challenges were further compounded by poverty, unemployment and low health literacy in some settings, which were viewed as key contributors to misuse and suboptimal care (Altayeb et al. 2025; Covvey et al. 2023; Mikhael et al. 2024).

Together, these accounts suggest that gabapentinoid misuse is rarely the result of a single cause. Rather, it emerges from the intersection of individual motivations, drug properties, healthcare system shortcomings and broader socioeconomic conditions.

#### 3.4.4 Potential Signs of Misuse and Dependence

The signs of misuse and dependence were discussed by participants in a number of studies (Buttram et al. 2017, 2019; Covvey et al. 2023; Jorgenson et al. 2021; Lehnus et al. 2024; Mikhael et al. 2024; Parbery‐Clark et al. 2025). Healthcare practitioners—particularly those working in substance use treatment settings—emphasised the need to remain vigilant to emerging patterns of misuse (e.g., “We always gotta stay ahead of the curve. If we see [gabapentin misuse] starting to develop, we gotta nip it”; Buttram et al. 2019). Healthcare practitioners noted that patients rarely disclosed misuse or dependence voluntarily due to stigma, legal concerns, or fears about losing access to treatment (e.g., “Patients don't want to intentionally reveal misusing drugs because… that could prevent them from getting said drugs to use, which hurts them,” Covvey et al. 2023). As a result, HCPs looked for indirect markers. Pharmacists cited expedited prescription refills (“The biggest sign in the pharmacy setting is obviously the early refill,” Covvey et al. 2023), unsanctioned dose escalations (“Sometimes patients will report that they self‐go up in dose without permission from the prescriber,” Covvey et al. 2023) and visible signs of intoxication or withdrawal (“Such customers are usually agitated,” Mikhael et al. 2024). Medical specialists reported additional red flags, including persistent requests for gabapentinoids, reported tolerance, reluctance to reduce or discontinue use and having more than three prescribers (Covvey et al. 2023; Jorgenson et al. 2021). Veterinarians reported similar behaviours in human clients misusing veterinary medicines: early refills, requests from multiple practices and specific drug demands (Lehnus et al. 2024). Drug diversion investigators also reported awareness of these signs of misuse based on their conversations with HCPs (e.g., “Physicians have reported numerous requests for early refills”; Buttram et al. 2017). In primary care, some prescribers use urine toxicology to detect non‐adherence or polysubstance use, often leading to discontinuation on safety grounds (Parbery‐Clark et al. 2025).

Participants commonly relied on behavioural and prescribing patterns—such as early refills, dose escalation, or patient agitation—as key indicators of potential misuse or dependence, particularly in the absence of direct disclosure.

#### 3.4.5 Navigating Ethical Dilemmas in Prescribing and Dispensing

Participants across several studies described the ethical and clinical dilemmas involved in prescribing and dispensing gabapentinoids, particularly in the context of chronic pain, substance use treatment and socioeconomic disadvantage (Buttram et al. 2019; Covvey et al. 2023; Ghinea et al. 2015; Mikhael et al. 2024; Parbery‐Clark et al. 2025). Medical specialists often felt torn between their duty to relieve suffering and their responsibility to minimise harm. As one explained, “There are people who are really in pain and […] we want to help and we have the power of being able to prescribe and give them things, and it's hard not to” (Parbery‐Clark et al. 2025). This tension was particularly acute in substance use treatment settings, where gabapentin was seen as helpful for managing withdrawal symptoms, anxiety and pain—factors that could otherwise hinder engagement (e.g., “It's a very touchy subject, when addicts have pain in recovery… I say, why should addicts and alcoholics be miserable?” Buttram et al. 2019). Yet concerns about misuse and a lack of clear prescribing guidance made decisions fraught. As one clinical director reflected, “There's no clear‐cut answer. I'd love to hear somebody say there is” (Buttram et al. 2019).

Medical specialists working in general practice also described challenges around shared decision‐making and patient agency (Covvey et al. 2023; Parbery‐Clark et al. 2025). They worried withholding medication could undermine therapeutic relationships or leave patients without adequate symptom relief. One medical specialist reflected, “Often we've created this problem, it's not the patient's fault” (Parbery‐Clark et al. 2025). Others feared more extreme consequences of deprescribing, including illicit sourcing and threats of self‐harm (Parbery‐Clark et al. 2025). Some resorted to forced reductions in cases of life‐threatening risk or suspected diversion (Parbery‐Clark et al. 2025). Dilemmas were heightened when alternative treatments were inaccessible due to cost or availability. As one physician assistant noted, the alternative was to suggest a referral “to a specialist that I know you can't afford” (Covvey et al. 2023).

Community pharmacists faced similar pressures (Mikhael et al. 2024). When misuse was suspected, some avoided confrontation by claiming the medication was out of stock (e.g., “I don't want to show him directly that I don't want to give him treatment. Instead I tell him I don't have the drug or the stock is just finished,” Mikhael et al. 2024). Nevertheless, the presence of a valid prescription often left pharmacists feeling obliged to dispense.

Across settings, professionals described navigating these dilemmas in the absence of clear guidance or adequate support, relying on personal judgement to balance ethical, clinical and relational concerns.

#### 3.4.6 Strategies for Prevention and Harm Reduction

Professionals across healthcare, policy and enforcement domains proposed a range of strategies to mitigate gabapentinoid misuse, dependence and associated harms. These strategies, outlined below, fell into four interrelated categories: improving clinical decision‐making around prescribing; strengthening regulatory and enforcement responses; expanding access to care for pain, distress and dependence and raising awareness through education and drug literacy initiatives.

##### 3.4.6.1 Improving Clinical Decision‐Making Around Prescribing

Professionals across diverse healthcare settings identified several opportunities to strengthen clinical decision‐making around gabapentinoid prescribing (Altayeb et al. 2025; Buttram et al. 2019; Coombes and Cooper 2019; Covvey et al. 2023; Gittins 2024; Jeffries et al. 2025; Jorgenson et al. 2021; Mikhael et al. 2024; Parbery‐Clark et al. 2025). These focused on improving individual practitioner judgement, clarifying clinical guidance, enhancing monitoring systems and strengthening interdisciplinary communication and collaboration.

Clinical uncertainty and gaps in guidance were key concerns. In both primary and specialist care, professionals described a lack of clear protocols for initiating, reviewing, tapering, or discontinuing gabapentinoids (Buttram et al. 2019; Coombes and Cooper 2019; Gittins 2024; Parbery‐Clark et al. 2025). In the UK, medical specialists reported that despite updated prescribing guidelines, they often felt ill‐equipped to manage dose reductions or navigate patient resistance to deprescribing (Parbery‐Clark et al. 2025). At the same time, a recovery worker expressed frustration at the lack of guidance on managing dependence: “In terms of stuff like pregabalin, there isn't anything basically… we haven't got a pathway to how we would deal with that” (Coombes and Cooper 2019).

Participants emphasised the importance of early‐stage prescribing decisions in shaping long‐term outcomes. Professionals recommended limiting the initiation of gabapentinoids whenever possible and, where prescribing was necessary, starting at low doses and framing the treatment as short‐term (Parbery‐Clark et al. 2025). These precautions were considered particularly important for patients with high‐risk features such as a history of substance use, psychiatric comorbidities, or previous prescription drug misuse (Parbery‐Clark et al. 2025).

Education and training were consistently identified as priorities across settings. Across multiple studies, HCPs called for more education on the risks of gabapentinoids and safe prescribing practices (Altayeb et al. 2025; Gittins 2024; Mikhael et al. 2024; Covvey et al. 2023). Community pharmacists in Iraq advocated for mandatory courses on managing patients experiencing misuse and dependence (Mikhael et al. 2024), while Sudanese pharmacists wanted more training on how to detect fraudulent prescriptions (Altayeb et al. 2025). In the US and UK, HCPs stressed the need for practical, up‐to‐date resources on tapering, drug interactions and harm reduction interventions (Covvey et al. 2023; Gittins 2024). Education was seen as an opportunity to raise awareness among prescribers about potential risks, with some suggesting it should occur through continued education requirements (Covvey et al. 2023). A community pharmacist noted, “I definitely think education would be good,” although some participants expressed scepticism about whether education alone would change prescribing behaviour (Covvey et al. 2023).

In addition to individual‐level learning, participants highlighted the importance of enhanced systems for prescribing oversight. Pharmacists in Sudan called for closer monitoring of pregabalin prescribing (Altayeb et al. 2025). In the US, attention focused on prescription drug monitoring programs (PDMPs). While PDMPs were widely viewed as useful, their impact was often undermined by inconsistent use, jurisdictional variation and limited enforcement (Covvey et al. 2023). As one community pharmacist noted, “You only have to do it if you feel the need to. And you can't even be sanctioned for misuse or bad professional judgment for not looking at it” (Covvey et al. 2023). Calls were made for more uniform implementation, real‐time data sharing and mandatory use policies (Covvey et al. 2023).

Participants also identified other tools to guide prescribing decisions. In UK prison settings, dashboards and prescribing safety indicators were used to flag potentially harmful combinations, such as co‐prescription of gabapentinoids with opioids (Jeffries et al. 2025). In Canada, prescribers responded positively to an audit and feedback tool that provided personalised prescribing data and comparisons with peers, encouraging reflection and behaviour change (Jorgenson et al. 2021).

Finally, improving clinical decision‐making was seen to depend not only on individual skills or tools, but also on interdisciplinary collaboration (Gittins 2024). Participants advocated for better communication between prescribers, pharmacists and treatment services, including shared training, coordinated care plans and regular communication (e.g., phone calls, letters) to support more consistent and accountable prescribing practices (Gittins 2024).

Professionals reported that improving decision‐making required not only better guidance and monitoring tools, but also a cultural shift toward more collaborative, evidence‐informed prescribing practices.

##### 3.4.6.2 Strengthening Regulatory and Enforcement Responses

Participants highlighted the role of regulatory and enforcement responses in mitigating gabapentinoid misuse and dependence (Altayeb et al. 2025; Buttram et al. 2023; Covvey et al. 2023; Mikhael et al. 2024; Mohamed and Bashir 2024; Jorgenson et al. 2021). Proposed strategies ranged from strengthening prescribing and dispensing controls to revising drug legislation, increasing market surveillance and expanding the remit of law enforcement.

Pharmacists in Sudan and Iraq called for stricter adherence to existing dispensing regulations, greater governmental surveillance, enforcement of laws and tighter restrictions on both prescribing and dispensing practices (Altayeb et al. 2025; Mikhael et al. 2024). In Somalia, participants emphasised that regulatory authorities should play a central role in ensuring that pharmaceutical markets are properly monitored and controlled (Mohamed and Bashir 2024). Updating and reforming existing drug legislation was also seen as essential to align regulatory frameworks with emerging public health needs (Mohamed and Bashir 2024). The role of law enforcement, professionals in Somalia suggested, should extend beyond punitive measures to include community engagement and support (Mohamed and Bashir 2024). This could include community policing and programs that refer individuals with substance use issues to support services (Mohamed and Bashir 2024). However, some professionals voiced concerns about regulatory measures that focused primarily on surveillance or monitoring. In particular, some Canadian medical specialists were worried that strategies to track prescribing could be perceived as punitive or threatening (Jorgenson et al. 2021).

Much of the debate around regulation was focused on the up‐scheduling of gabapentinoids. Among US law enforcement officers, support for scheduling was widespread, particularly in jurisdictions where gabapentin had already been classified as a controlled substance (Buttram et al. 2023). Officers in these states consistently viewed scheduling as necessary to curb misuse and diversion (e.g., “We have seen the abuse rate soar until it was listed as a Schedule V”) and believed it had helped deter inappropriate prescribing (e.g., “I feel doctors feel comfortable prescribing gabapentin… because it is not listed as a [controlled substance]”) and dispensing (e.g., “Pharmacists don't blink at filling non‐controlled prescriptions”). Others felt the impact on misuse was limited (e.g., *“*not much of a deterrent for abusers”) but still supported scheduling for symbolic or operational reasons (e.g., to facilitate data collection, prevent deaths from suicide, or ensure harsher penalties for black market sales).

Healthcare practitioners and drug policy experts in the US expressed more mixed views on scheduling. Some acknowledged modest benefits to patient safety because prescribers were required to be “more responsible with those prescriptions” (Covvey et al. 2023). However, others raised concerns that scheduling could restrict access for people using gabapentinoids appropriately, particularly those with limited treatment alternatives (e.g., “I hate to even think about how that may vary state‐to‐state and create issues for access to the patients who are benefiting from it*”*; Covvey et al. 2023). Several HCPs and drug policy experts questioned the overall effectiveness of scheduling as a deterrent, citing parallels with opioid regulation: “We're going to see exactly what happened, which I and some of the other drug policy scholars predicted would happen, when states began adopting legislation that limited opioid prescribing – there was going to be an increase in the number of people who started going to the illicit supply” (Covvey et al. 2023).

There was also concern that scheduling could increase HCP stigma toward patients and hamper shared decision‐making. A pharmacist and drug policy expert believed that HCPs supporting scheduling were either looking for an easy excuse not to prescribe the drug in question or had “a desire to police as opposed to a desire to improve or change their daily practice”. Drug policy experts were generally critical of any regulatory efforts to mitigate misuse that could have legal ramifications for patients, with one describing increased regulation as a “*band‐aid*” solution to the public health challenge (Covvey et al. 2023).

Overall, while regulatory and enforcement measures were seen as potentially important tools, participants emphasised the need for balanced approaches that protect public health without creating unintended harms or exacerbating inequities.

##### 3.4.6.3 Expanding Access to Care for Dependence, Pain and Distress

Healthcare practitioners emphasised that a key strategy for preventing and reducing harm related to gabapentinoid misuse and dependence lies in expanding access to comprehensive, person‐centred care for individuals experiencing substance dependence, chronic pain and psychological distress (Altayeb et al. 2025; Coombes and Cooper 2019; Covvey et al. 2023; Gittins 2024; Mohamed and Bashir 2024; Parbery‐Clark et al. 2025). This would involve not only increasing the availability of services but also addressing the structural, cultural and systemic barriers that hinder engagement with care.

Community pharmacists and HCPs working in substance use treatment settings highlighted the need to broaden treatment and rehabilitation services, particularly for individuals dependent on prescription drugs (Altayeb et al. 2025; Coombes and Cooper 2019; Mohamed and Bashir 2024). Participants recommended the development of specialist services and care pathways specifically tailored to prescription drug dependence, distinct from those designed for illicit drug use (Coombes and Cooper 2019). The roles of social workers and family support services in substance use treatment were also emphasised (Mohamed and Bashir 2024), particularly in providing assessment, counselling, coordinated care, advocacy and addressing the broader social dynamics that impact engagement and long‐term outcomes.

The integration of mental health care was similarly positioned as a cornerstone of prevention and harm reduction (Mohamed and Bashir 2024; Parbery‐Clark et al. 2025). Untreated psychological distress was recognised as a key driver of both substance use and chronic pain, with participants stressing the need to expand access to psychological therapies to reduce reliance on pharmacological coping strategies.

Improving access to non‐pharmacological pain treatments was seen as critical to mitigating gabapentinoid misuse and dependence (Buttram et al. 2019; Covvey et al. 2023; Ghinea et al. 2015; Gittins 2024; Parbery‐Clark et al. 2025). Many HCPs noted that gabapentinoids were often prescribed off‐label in cases where there were better available treatments (Buttram et al. 2019; Covvey et al. 2023; Ghinea et al. 2015). Despite the potential benefits of non‐pharmacological approaches, barriers such as affordability, limited availability, short appointment times, low prescriber confidence and poor patient engagement often restricted their use (Covvey et al. 2023; Buttram et al. 2019; Ghinea et al. 2015; Parbery‐Clark et al. 2025). A community pharmacist in the US noted that, “getting quality physicians to go into pain management as a speciality can just be daunting for doctors to go that route because of the liability and everything with it” (Covvey et al. 2023). Promoting inclusive, non‐stigmatising healthcare environments—particularly for individuals with a history of substance use or from racially minoritised backgrounds—was also identified as essential for improving access, encouraging help‐seeking and ensuring quality care (Covvey et al. 2023).

Participants further explained that improving the quality of chronic pain care required both structural reform and cultural change, including recalibrating patient and prescriber expectations around treatment outcomes (Covvey et al. 2023; Parbery‐Clark et al. 2025). Emphasis was placed on demedicalising chronic pain and focusing on functional rather than purely symptomatic improvements (Covvey et al. 2023; Gittins 2024; Parbery‐Clark et al. 2025). As one prescriber in the UK stated, “we can't make pain go away. We can make the suffering go, but living with pain is something that we need to focus on” (Parbery‐Clark et al. 2025), underscoring the need to reframe chronic pain as a condition to be managed rather than cured.

Across studies, participants highlighted the need for a multi‐faceted response to gabapentinoid misuse and dependence that extends beyond individual prescribing practices. Suggested approaches included investing in accessible, non‐pharmacological care options, developing tailored support for prescription drug dependence, reducing stigma within healthcare and shifting cultural narratives around pain. In the absence of such systemic changes, some participants noted that prescribers felt limited to suboptimal pharmacological choices, while individuals seeking relief were more likely to engage in potentially harmful use.

##### 3.4.6.4 Raising Awareness and Building Drug Literacy

Participants across multiple studies emphasised the need to raise awareness of harms associated with gabapentinoid misuse and dependence among both patients and the public more broadly (Altayeb et al. 2025; Coombes and Cooper 2019; Falzon et al. 2023; Gittins 2024; Mohamed and Bashir 2024; Parbery‐Clark et al. 2025). Awareness was framed not only as a means of reducing stigma and enabling earlier identification and treatment, but also as critical to fostering safer, more informed use (Coombes and Cooper 2019; Gittins 2024; Mohamed and Bashir 2024). Some advocated for more consistent and proactive patient education at the point of prescribing and dispensing, including clear explanations of rationale and risk (Covvey et al. 2023; Parbery‐Clark et al. 2025). Others called for targeted outreach in primary care and substance use services (e.g., “We need a much more aggressive outreach service going into primary care which helps people understand dependence on prescribed medication, which helps people to seek help, which helps people take control of their prescription and reduce it themselves,” Coombes and Cooper 2019). However, implementation was seen as difficult in under‐resourced clinical settings (e.g., “Raising the profile more within the service, posters and leaflets… tends to be lower on the list of priorities,” Gittins 2024). There was also uncertainty about who should lead messaging efforts: “There is a need for the message to get out there, somehow, yeah but where the message comes from I don't know” (Coombes and Cooper 2019).

Public‐facing campaigns were also recommended (Altayeb et al. 2025; Mohamed and Bashir 2024), with participants urging greater leadership from national authorities and community leaders. Community‐based initiatives—including those in schools and faith settings—were seen as crucial for reaching at‐risk groups and reshaping cultural norms (Mohamed and Bashir 2024). Finally, the drug checking services were seen as an opportunity to foster personalised, real‐time conversations about risk (Falzon et al. 2023).

These perspectives reflect a broad consensus that raising awareness—through coordinated efforts across clinical, community and policy settings—is essential to reducing harm and fostering more careful, considered use of gabapentinoids.

## 4 Discussion

The aim of this qualitative systematic review was to explore the perspectives of HCPs and other professional groups on the topic of gabapentinoid misuse and dependence. We located 19 relevant studies, most of which were conducted in North America or Europe. All included studies were published within the last decade, with most appearing since 2023, reflecting the rapidly evolving nature of this literature. No studies were identified on mirogabalin, a newer gabapentinoid approved in several Asian countries (Tang et al. 2023).

The professionals whose perspectives were synthesised in this review included HCPs, law enforcement and regulatory personnel, drug policy experts, teachers, religious leaders and veterinarians. There was considerable variability both within and between these professional groups regarding awareness of gabapentinoid misuse and dependence and perceptions of the associated risks. Law enforcement officers were generally more concerned about gabapentinoid‐related harms than HCPs, possibly due to their firsthand experiences of responding to fatal overdoses and suicides. In addition, law enforcement officers might have more frequent exposure to marginalised populations that are less likely or unable to access medical services, which may contribute to a bias toward observing harms over therapeutic benefits. Many HCPs viewed gabapentinoids as a comparatively safer alternative to opioids and described them as a preferred option in the context of regulatory scrutiny and growing concern over opioid‐related harms. This suggests that the rise in gabapentinoid prescribing reflects not only clinical judgement, but also broader ethical, legal and regulatory pressures to reduce opioid use (Goodman and Brett 2017).

The reasons for misuse identified by professionals—to alter mood or consciousness, to potentiate or substitute for opioids and to self‐medicate—map closely onto the reasons reported by those with lived experience of gabapentinoid misuse (Al‐Husseini et al. 2018; Buttram and Kurtz 2021; McNeilage, Ashton‐James, and Scholz 2023; McNeilage et al. 2024; Servais et al. 2023; Vickers Smith et al. 2018). This alignment suggests these professional groups have a good grasp of the factors contributing to misuse. Similarly, the signs of gabapentinoid misuse and dependence identified by HCPs in this review—such as expedited refills, dose escalations and resistance to dose reductions—correspond with the existing literature on indicators of prescription drug misuse (Portenoy 1996; Webster and Webster 2005), suggesting strategies developed in other contexts to help prescribers identify at‐risk patients may have relevance here (Klimas et al. 2019).

Prescriber uncertainty was a recurring theme, with many professionals citing a lack of clinical guidance for initiating, monitoring, or tapering gabapentinoid treatment. This uncertainty, coupled with structural constraints such as limited access to non‐pharmacological alternatives, contributed to inconsistent or suboptimal prescribing practices. Although many called for improved education and clinical training, there was also recognition that knowledge alone is unlikely to shift entrenched prescribing habits without systemic reforms and cultural change.

Finally, professionals proposed a wide range of strategies to reduce gabapentinoid‐related harms, including prescriber education, enhanced regulatory measures, improved access to care for pain and dependence and expanded drug literacy through clinical and community‐based education. In considering these approaches, policymakers should draw on lessons from responses to the “opioid crisis” and remain mindful of the potential for unintended consequences, including increased stigma, reduced access or diversion to illicit markets (Ansari et al. 2020; Furlan et al. 2018; Haegerich et al. 2019).

### 4.1 Implications for Prescribers, Policymakers and Researchers

A number of HCPs reported limited awareness of, and concern for, gabapentinoid misuse and dependence, particularly in primary care settings. Prescribers were generally supportive of the need for greater education in this area, despite some scepticism that knowledge alone would change influence prescriber behaviour. Several ongoing research projects are examining patient and prescriber education initiatives to improve knowledge about gabapentinoids and promote safe and appropriate prescribing (Cole et al. 2021; Collinson 2019; NCT04294901; NCT04628832). The findings of these studies will shed light on the likely effectiveness of these approaches. Regardless, deprescribing should be considered where there is an unfavourable benefit‐to‐harm ratio (Reeve et al. 2017). Given gabapentinoids can be associated with misuse, dependence and withdrawal, deprescribing should be gradual, patient‐centred and well‐supported. Unfortunately, a recent scoping review found insufficient evidence to guide best practice in this area (Anderson et al. 2023), highlighting an urgent need for high‐quality research to inform safe and effective deprescribing protocols.

Professionals across several studies drew a clear connection between the rise in gabapentinoid misuse and the tightening of opioid regulations. Gabapentinoids were said to be a popular substitute for opioids because they were less regulated, more readily available and less likely to appear on drug screens. Healthcare practitioners also reported cases of patients turning to gabapentinoid misuse following a forced opioid taper or because, unlike opioids, the effects were not blocked by buprenorphine. Given that the increased regulation of opioids appears to be contributing to gabapentinoid misuse, efforts to restrict the supply of gabapentinoids should be mindful of the possibility of causing substitution with a different, potentially more harmful, substance. At the same time, it is important to safeguard access to gabapentinoids for patients when therapeutically appropriate.

When discussing strategies to curb gabapentinoid misuse and dependence, HCPs emphasised the need to address the root causes of overprescription. They explained that systemic and cultural barriers to nonpharmacological treatment had led to an overreliance on medication for chronic pain. The reported barriers to multidisciplinary and nonpharmacological care included a lack of prescriber time and training, a lack of service availability, particularly in rural areas and a lack of affordability and insurance coverage, particularly in countries without universal healthcare. In addition, cultural expectations around pain—in particular, the expectation for immediate and complete pain relief—meant that engaging patients in nonpharmacological strategies could be challenging. For prescribers working within these constraints, writing a prescription was often the fastest and most cost‐effective option for patients. As the push away from opioids has gained momentum, it seems gabapentinoids have filled some of the treatment gap. If restrictions on gabapentinoids are implemented without parallel investment in non‐pharmacological options, there is a real risk of therapeutic gaps being filled by other potentially harmful drugs.

### 4.2 Limitations

There are several limitations to consider when interpreting the findings of this review. First, the majority of included studies were conducted in the US and UK, and the transferability of these findings to settings with different regulatory environments, health system structures and sociocultural contexts remains unclear. Patterns of gabapentinoid prescribing and misuse are likely to vary internationally, and there is comparatively little published evidence on the prevalence or drivers of misuse in other regions. Further research is needed to address this gap and explore how local contextual factors shape gabapentinoid‐related harms and responses across diverse settings. It is also worth noting that the majority of the included studies were conducted in substance use treatment settings and law enforcement‐related settings (e.g., drug diversion investigators, drug courts, prisons), where participants may be more likely to encounter individuals at higher risk of misuse and dependence—such as those with psychiatric comorbidities or a history of substance use disorders. Patients prescribed gabapentinoids for chronic pain, without these additional risk factors, may be less likely to develop misuse or dependence. More research is needed in primary care and other generalist settings to better understand patterns of use, perceptions of risk and dependence‐related behaviours in patients using gabapentinoids as prescribed. Nevertheless, Table 1 provides descriptions of the samples and settings, allowing readers to assess the relevance of the current evidence to their own contexts. Second, only six studies had direct relevance to the review question, three of which came from the same research group, potentially biasing the way the issue was presented. Third, although our synthesis included nine review findings, only two were assessed as high confidence, five as moderate and two as low using the GRADE‐CERQual approach. These lower ratings were mainly due to limited data depth, indirect relevance to gabapentinoids and narrow geographic representation. While the methodological quality of many studies was acceptable, the thin or context‐specific nature of the data highlights the need for stronger qualitative research. Future studies should aim for richer, more focused data, broader representation across professional roles and contexts and improved methodological transparency, including clearer reporting of theoretical frameworks and researcher reflexivity.

## 5 Conclusion

This systematic review highlights the complex and evolving nature of gabapentinoid misuse and dependence, as understood by HCPs and other professional groups. While gabapentinoids were often framed as a safer alternative to opioids, professionals expressed growing unease about suboptimal prescribing and associated patient harms. These concerns were commonly linked to structural issues within healthcare systems—most notably, poor access to non‐pharmacological pain management and the downstream effects of opioid deprescribing. Prevention and harm reduction strategies must go beyond individual prescribers to tackle the broader social, cultural and systemic drivers of misuse, while also avoiding the unintended consequences of overregulation. A balanced, compassionate and evidence‐informed approach is critical. At the same time, it is important to situate gabapentinoid‐related harms within the broader context of psychoactive drug use. Other psychoactive substances –such as benzodiazepines, stimulants and some antidepressants—also carry risks of misuse, dependence and in some cases greater harm. While concern about gabapentinoids is justified, responses should remain proportionate and avoid unintended consequences by considering the wider spectrum of drug‐related risks.

## Author Contributions

**Amy G. McNeilage:** conceptualisation (lead), data curation (lead), formal analysis (lead), funding acquisition (equal), investigation (lead), methodology (lead), project administration (lead), visualisation (lead), writing – original draft (lead), and writing – review and editing (supporting). **Evan Browne:** conceptualisation (supporting), data curation (supporting), formal analysis (supporting), investigation (supporting), methodology (supporting), project administration (supporting), visualisation (supporting), writing – original draft (supporting), and writing – review and editing (lead). **Suzanne Nielsen:** conceptualisation (supporting), funding acquisition (equal), investigation (supporting), methodology (supporting), supervision (supporting), and writing – review and editing (supporting). **Claire E. Ashton‐James:** conceptualisation (supporting), funding acquisition (equal), methodology (supporting), supervision (supporting), and writing – review and editing (supporting). **Bridin Murnion:** conceptualisation (supporting), investigation (supporting), methodology (supporting), supervision (lead), and writing – review and editing (supporting).

## Conflicts of Interest

The authors declare no conflicts of interest.

## Supporting information


**Data S1:** ejp70116‐sup‐0001‐supinfo.docx.
