# Supplementary material for: Healthcare Practitioner and Other Professionals' Perspectives on Gabapentinoid Misuse and Dependence: A Systematic Review of Qualitative Studies
Source: Eur J Pain. 2025 Sep 2;29(9):e70116. doi: 10.1002/ejp.70116 (PMC12402873; doi:10.1002/ejp.70116)
Supplement: Supplementary file 1 — Data S1: ejp70116‐sup‐0001‐supinfo.docx. [file EJP-29-0-s001.docx]

**Supplementary Table 1.** Completed PRISMA checklist

| **Section and Topic** | **Item #** | **Checklist item** | **Location where item is reported** |
| --- | --- | --- | --- |
| **TITLE** | | |  |
| Title | 1 | Identify the report as a systematic review. | Page 1 |
| **ABSTRACT** | | |  |
| Abstract | 2 | See the PRISMA 2020 for Abstracts checklist. | Abstract |
| **INTRODUCTION** | | |  |
| Rationale | 3 | Describe the rationale for the review in the context of existing knowledge. | Page 2 (Intro.) |
| Objectives | 4 | Provide an explicit statement of the objective(s) or question(s) the review addresses. | Page 2 (Intro.) |
| **METHODS** | | |  |
| Eligibility criteria | 5 | Specify the inclusion and exclusion criteria for the review and how studies were grouped for the syntheses. | Page 3 (2.1.) |
| Information sources | 6 | Specify all databases, registers, websites, organisations, reference lists and other sources searched or consulted to identify studies. Specify the date when each source was last searched or consulted. | Page 4 (2.2.) |
| Search strategy | 7 | Present the full search strategies for all databases, registers and websites, including any filters and limits used. | Supp. |
| Selection process | 8 | Specify the methods used to decide whether a study met the inclusion criteria of the review, including how many reviewers screened each record and each report retrieved, whether they worked independently, and if applicable, details of automation tools used in the process. | Pages 4,5 (2.3.) |
| Data collection process | 9 | Specify the methods used to collect data from reports, including how many reviewers collected data from each report, whether they worked independently, any processes for obtaining or confirming data from study investigators, and if applicable, details of automation tools used in the process. | Page 5 (2.4.) |
| Data items | 10a | List and define all outcomes for which data were sought. Specify whether all results that were compatible with each outcome domain in each study were sought (e.g. for all measures, time points, analyses), and if not, the methods used to decide which results to collect. | NA |
|  | 10b | List and define all other variables for which data were sought (e.g. participant and intervention characteristics, funding sources). Describe any assumptions made about any missing or unclear information. | NA |
| Study risk of bias assessment | 11 | Specify the methods used to assess risk of bias in the included studies, including details of the tool(s) used, how many reviewers assessed each study and whether they worked independently, and if applicable, details of automation tools used in the process. | Page 5 (2.5.) |
| Effect measures | 12 | Specify for each outcome the effect measure(s) (e.g. risk ratio, mean difference) used in the synthesis or presentation of results. | NA |
| Synthesis methods | 13a | Describe the processes used to decide which studies were eligible for each synthesis (e.g. tabulating the study intervention characteristics and comparing against the planned groups for each synthesis (item #5)). | NA |
|  | 13b | Describe any methods required to prepare the data for presentation or synthesis, such as handling of missing summary statistics, or data conversions. | NA |
|  | 13c | Describe any methods used to tabulate or visually display results of individual studies and syntheses. | NA |
|  | 13d | Describe any methods used to synthesize results and provide a rationale for the choice(s). If meta-analysis was performed, describe the model(s), method(s) to identify the presence and extent of statistical heterogeneity, and software package(s) used. | Pages 5, 6 (2.6.) |
|  | 13e | Describe any methods used to explore possible causes of heterogeneity among study results (e.g. subgroup analysis, meta-regression). | NA |
|  | 13f | Describe any sensitivity analyses conducted to assess robustness of the synthesized results. | NA |
| Reporting bias assessment | 14 | Describe any methods used to assess risk of bias due to missing results in a synthesis (arising from reporting biases). | NA |
| Certainty assessment | 15 | Describe any methods used to assess certainty (or confidence) in the body of evidence for an outcome. | Page 6 (2.7.) |
| **RESULTS** | | |  |
| Study selection | 16a | Describe the results of the search and selection process, from the number of records identified in the search to the number of studies included in the review, ideally using a flow diagram. | Fig. 1 |
|  | 16b | Cite studies that might appear to meet the inclusion criteria, but which were excluded, and explain why they were excluded. | Supp. |
| Study characteristics | 17 | Cite each included study and present its characteristics. | T1 |
| Risk of bias in studies | 18 | Present assessments of risk of bias for each included study. | T1, Supp. |
| Results of individual studies | 19 | For all outcomes, present, for each study: (a) summary statistics for each group (where appropriate) and (b) an effect estimate and its precision (e.g. confidence/credible interval), ideally using structured tables or plots. | NA |
| Results of syntheses | 20a | For each synthesis, briefly summarise the characteristics and risk of bias among contributing studies. | T2 |
|  | 20b | Present results of all statistical syntheses conducted. If meta-analysis was done, present for each the summary estimate and its precision (e.g. confidence/credible interval) and measures of statistical heterogeneity. If comparing groups, describe the direction of the effect. | NA |
|  | 20c | Present results of all investigations of possible causes of heterogeneity among study results. | NA |
|  | 20d | Present results of all sensitivity analyses conducted to assess the robustness of the synthesized results. | NA |
| Reporting biases | 21 | Present assessments of risk of bias due to missing results (arising from reporting biases) for each synthesis assessed. | NA |
| Certainty of evidence | 22 | Present assessments of certainty (or confidence) in the body of evidence for each outcome assessed. | Page 9 (3.4.1) |
| **DISCUSSION** | | |  |
| Discussion | 23a | Provide a general interpretation of the results in the context of other evidence. | Pages 25-26 |
|  | 23b | Discuss any limitations of the evidence included in the review. | Pages 28-29 (4.2.) |
|  | 23c | Discuss any limitations of the review processes used. | Pages 28-29 (4.2.) |
|  | 23d | Discuss implications of the results for practice, policy, and future research. | Pages 26-28 (4.1.) |
| **OTHER INFORMATION** | | |  |
| Registration and protocol | 24a | Provide registration information for the review, including register name and registration number, or state that the review was not registered. | Page 3 (2.) |
|  | 24b | Indicate where the review protocol can be accessed, or state that a protocol was not prepared. | Page 3 (2.) |
|  | 24c | Describe and explain any amendments to information provided at registration or in the protocol. | Page 7 (2.9.) |
| Support | 25 | Describe sources of financial or non-financial support for the review, and the role of the funders or sponsors in the review. | Title page |
| Competing interests | 26 | Declare any competing interests of review authors. | Title page |
| Availability of data, code and other materials | 27 | Report which of the following are publicly available and where they can be found: template data collection forms; data extracted from included studies; data used for all analyses; analytic code; any other materials used in the review. | Title page |

**Supplementary Table 2.** Completed ENTREQ checklist

| **No** | **Item** | **Guide and description** | **Reported on page** |
| --- | --- | --- | --- |
| **1** | Aim | State the research question the synthesis addresses. | 2 |
| **2** | Synthesis methodology | Identify the synthesis methodology or theoretical framework which underpins the synthesis, and describe the rationale for choice of methodology *(e.g. meta-ethnography, thematic synthesis, critical interpretive synthesis, grounded theory synthesis, realist synthesis, meta-aggregation, meta-study, framework synthesis).* | 5,6 |
| **3** | Approach to searching | Indicate whether the search was pre-planned (*comprehensive search strategies to seek all available studies)* or iterative (*to seek all available concepts until they theoretical saturation is achieved)*. | 4 |
| **4** | Inclusion criteria | Specify the inclusion/exclusion criteria *(e.g. in terms of population, language, year limits, type of publication, study type).* | 3 |
| **5** | Data sources | Describe the information sources used (e.g. *electronic databases (MEDLINE, EMBASE, CINAHL, psycINFO, Econlit), grey literature databases (digital thesis, policy reports), relevant organisational websites, experts, information specialists, generic web searches (Google Scholar) hand searching, reference lists)* and when the searches conducted; provide the rationale for using the data sources. | 4 |
| **6** | Electronic Search strategy | Describe the literature search *(e.g. provide electronic search strategies with population terms, clinical or health topic terms, experiential or social phenomena related terms, filters for qualitative research, and search limits)*. | 4, Supp. |
| **7** | Study screening methods | Describe the process of study screening and sifting *(e.g. title, abstract and full text review, number of independent reviewers who screened studies).* | 4,5 |
| **8** | Study characteristics | Present the characteristics of the included studies *(e.g. year of publication, country, population, number of participants, data collection, methodology, analysis, research questions).* | T1 |
| **9** | Study selection results | Identify the number of studies screened and provide reasons for study exclusion *(e,g, for comprehensive searching, provide numbers of studies screened and reasons for exclusion indicated in a figure/flowchart; for iterative searching describe reasons for study exclusion and inclusion based on modifications t the research question and/or contribution to theory development).* | F1 |
| **10** | Rationale for appraisal | Describe the rationale and approach used to appraise the included studies or selected findings *(e.g. assessment of conduct (validity and robustness), assessment of reporting (transparency), assessment of content and utility of the findings).* | 5 |
| **11** | Appraisal items | State the tools, frameworks and criteria used to appraise the studies or selected findings *(e.g. Existing tools: CASP, QARI, COREQ, Mays and Pope* [[25](https://bmcmedresmethodol.biomedcentral.com/articles/10.1186/1471-2288-12-181#ref-CR25)]*; reviewer developed tools; describe the domains assessed: research team, study design, data analysis and interpretations, reporting).* | 5 |
| **12** | Appraisal process | Indicate whether the appraisal was conducted independently by more than one reviewer and if consensus was required. | 5 |
| **13** | Appraisal results | Present results of the quality assessment and indicate which articles, if any, were weighted/excluded based on the assessment and give the rationale. | 5, 8, T1, Supp. |
| **14** | Data extraction | Indicate which sections of the primary studies were analysed and how were the data extracted from the primary studies? *(e.g. all text under the headings “results /conclusions” were extracted electronically and entered into a computer software).* | 5 |
| **15** | Software | State the computer software used, if any. | 4, 5, 6 |
| **16** | Number of reviewers | Identify who was involved in coding and analysis. | 6 |
| **17** | Coding | Describe the process for coding of data *(e.g. line by line coding to search for concepts).* | 5, 6 |
| **18** | Study comparison | Describe how were comparisons made within and across studies *(e.g. subsequent studies were coded into pre-existing concepts, and new concepts were created when deemed necessary).* | 6 |
| **19** | Derivation of themes | Explain whether the process of deriving the themes or constructs was inductive or deductive. | 6 |
| **20** | Quotations | Provide quotations from the primary studies to illustrate themes/constructs, and identify whether the quotations were participant quotations of the author’s interpretation. | 6, 10-24 |
| **21** | Synthesis output | Present rich, compelling and useful results that go beyond a summary of the primary studies (e.g. *new interpretation, models of evidence, conceptual models, analytical framework, development of a new theory or construct).* | 9-25 |

**Supplementary Table 3.** Medline search strategy

**MeSH Terms in bold**

1 gabapentinoid*.mp. 1121

2 gabapentin.mp. or **Gabapentin/** 8966

3 pregabalin.mp. or **Pregabalin/** 5260

4 lyrica.mp. 153

5 neurontin.mp. 189

6 mirogabalin.mp. 143

7 1 or 2 or 3 or 4 or 5 or 6 13140

8 misus*.mp. 36144

9 abus*.mp. 222607

10 nonmedical*.mp. or Prescription **Drug Misuse/** 9493

11 non-medical*.mp. 9722

12 nontherapeutic*.mp. 2407

13 non-therapeutic*.mp. 1221

14 extramedical*.mp. 59

15 extra-medical*.mp. 145

16 nonprescribed.mp. 301

17 non-prescribed.mp. 687

18 nonprescription*.mp. 8187

19 non-prescription*.mp. 1529

20 substance us*.mp. 62646

21 **Drug Users/** or drug us*.mp. 84903

22 illicit.mp. 31062

23 (harmful adj3 us*).mp. [mp=title, book title, abstract, original title, name of substance word, subject heading word, floating sub-heading word, keyword heading word, organism supplementary concept word, protocol supplementary concept word, rare disease supplementary concept word, unique identifier, synonyms, population supplementary concept word, anatomy supplementary concept word] 3629

24 (risky adj3 us*).mp. [mp=title, book title, abstract, original title, name of substance word, subject heading word, floating sub-heading word, keyword heading word, organism supplementary concept word, protocol supplementary concept word, rare disease supplementary concept word, unique identifier, synonyms, population supplementary concept word, anatomy supplementary concept word] 2309

25 (high-risk adj3 us*).mp. [mp=title, book title, abstract, original title, name of substance word, subject heading word, floating sub-heading word, keyword heading word, organism supplementary concept word, protocol supplementary concept word, rare disease supplementary concept word, unique identifier, synonyms, population supplementary concept word, anatomy supplementary concept word] 9217

26 (high risk adj3 us*).mp. [mp=title, book title, abstract, original title, name of substance word, subject heading word, floating sub-heading word, keyword heading word, organism supplementary concept word, protocol supplementary concept word, rare disease supplementary concept word, unique identifier, synonyms, population supplementary concept word, anatomy supplementary concept word] 9217

27 (hazardous adj3 us*).mp. [mp=title, book title, abstract, original title, name of substance word, subject heading word, floating sub-heading word, keyword heading word, organism supplementary concept word, protocol supplementary concept word, rare disease supplementary concept word, unique identifier, synonyms, population supplementary concept word, anatomy supplementary concept word] 3020

28 (unsanctioned adj3 us*).mp. [mp=title, book title, abstract, original title, name of substance word, subject heading word, floating sub-heading word, keyword heading word, organism supplementary concept word, protocol supplementary concept word, rare disease supplementary concept word, unique identifier, synonyms, population supplementary concept word, anatomy supplementary concept word] 32

29 aberrant.mp. 119680

30 nonadherent.mp. 5535

31 non-adherent.mp. 5220

32 nonadherence.mp. 8132

33 non-adherence.mp. 9139

34 non-compliant.mp. 2801

35 noncompliant.mp. 3080

36 non-compliance.mp. 6930

37 noncompliance.mp. 7558

38 divert*.mp. 52862

39 diversion.mp. 29858

40 street.mp. 17060

41 tamper*.mp. 2446

42 intoxicate*.mp. 9348

43 intoxication.mp. 55389

44 recreation*.mp. 46571

45 euphori*.mp. 4007

46 polydrug*.mp. 1973

47 **Illicit Drugs/** 13306

48 poly-drug*.mp. 477

49 **Dependency, Psychological/** or dependen*.mp. 2117111

50 **Behavior, Addictive/** or addict*.mp. or **Substance-Related Disorders/** 183931

51 withdraw*.mp. 164244

52 **Substance Withdrawal Syndrome/** 23314

53 toleran*.mp. 433299

54 **Craving/** or crav*.mp. 13470

55 habitua*.mp. 50090

56 detox*.mp. 60248

57 rehab*.mp. 414180

58 **Drug Overdose/** or overdose*.mp. 35314

59 wean*.mp. 66336

60 taper*.mp. 29712

61 discontinu*.mp. 169308

62 cessat*.mp. 107807

63 8 or 9 or 10 or 11 or 12 or 13 or 14 or 15 or 16 or 17 or 18 or 19 or 20 or 21 or 22 or 23 or 24 or 25 or 26 or 27 or 28 or 29 or 30 or 31 or 32 or 33 or 34 or 35 or 36 or 37 or 38 or 39 or 40 or 41 or 42 or 43 or 44 or 45 or 46 or 47 or 48 or 49 or 50 or 51 or 52 or 53 or 54 or 55 or 56 or 57 or 58 or 59 or 60 or 61 or 62 4150831

64 **Qualitative Research/** or qualitative.mp. 411804

65 interview*.mp. or **Interview/** 553895

66 **Focus Groups/** or focus group*.mp. 81785

67 grounded theory.mp. or **Grounded Theory/** 17409

68 thematic analysis.mp. 56906

69 mixed-method*.mp. 57427

70 **Surveys and Questionnaires/ 625513**

71 open-ended.mp. 24532

72 lived experience*.mp. 17143

73 phenomenology.mp. 13286

74 phenomenological.mp. 27786

75 phenomenography.mp. 281

76 phenomenographical.mp. 44

77 ethnography.mp. 5411

78 ethnographical.mp. 136

79 ethnology.mp. or **Ethnology/** 183099

80 ethnological.mp. 262

81 ethnomethodology.mp. 125

82 ethnomethodological.mp. 106

83 autoethnography.mp. 347

84 autoethnographical.mp. 15

85 narrativ*.mp. 111351

86 **Personal Narrative/** 6737

87 content analysis.mp. 53478

88 discourse analysis.mp. 3024

89 conversation analysis.mp. 1105

90 theme*.mp. 171558

91 thematic*.mp. 98101

92 reflexiv*.mp. 11936

93 audiorecord*.mp. 544

94 audio-record*.mp. 13880

95 videorecord*.mp. 592

96 video-record*.mp. 40257

97 videotap*.mp. 21331

98 video-tap*.mp. 1266

99 taperecord*.mp. 18

100 tape-record*.mp. 7140

101 64 or 65 or 66 or 67 or 68 or 69 or 70 or 71 or 72 or 73 or 74 or 75 or 76 or 77 or 78 or 79 or 80 or 81 or 82 or 83 or 84 or 85 or 86 or 87 or 88 or 89 or 90 or 91 or 92 or 93 or 94 or 95 or 96 or 97 or 98 or 99 or 100 1769242

102 7 and 63 and 101 189

**Supplementary Table 4.** CINAHL search strategy

("gabapentinoid*" OR (MH "Gabapentin") OR (MH "Pregabalin") OR "Pregabalin" OR "Gabapentin" OR "mirogabalin" OR "LYRICA" OR "neurontin")

AND

("misus*" OR (MH "Substance Abuse+") OR (MH "Drug Rehabilitation Programs+") OR "nonmedical*" OR "non-medical*" OR (MH "Street Drugs+") OR "non-therapeutic*" OR "extramedical*" OR "extra-medical*" OR "nonprescribed" OR (MH "Drugs, Non-Prescription") OR "nonprescription*" OR "non-prescription*" OR "substance us*" OR (MH "Substance Abusers+") OR (MH "Substance Use Disorders+") OR (MH "Substance Use Rehabilitation Programs+") OR "drug us*" OR (MH "Intravenous Drug Users") OR "illicit N3 us*" OR "risky" OR (MH "Harm Reduction") OR "harmful" OR "high-risk" OR "hazardous" OR "aberrant" OR "unsanctioned" OR "nonadheren*" OR "non-adheren*" OR "non-complian*" OR "noncomplian*" OR "diversion" OR "divert*" OR "street" OR "tamper*" OR "intoxicat*" OR (MH "Recreational Drug Use") OR "recreation*" OR "euphori*" OR "polydrug" OR "poly-drug" OR "dependen*" OR "addict*" OR (MH "Substance Withdrawal Syndrome+") OR "withdraw*" OR "toleran*" OR (MH "Craving") OR "crav*" OR "habitua*" OR "detox*" OR "rehab*" OR (MH "Overdose+") OR "overdos*" OR (MH "Drug Tapering") OR "wean*" OR "taper*" OR (MH "Deprescribing") OR "discontinu*" OR "cessat*")

AND

((MH "Qualitative Studies+") OR (MH "Reflexivity (Research)") OR (MH "Phenomenology") OR (MH "Multimethod Studies") OR (MH "Semi-Structured Interview") OR (MH "Unstructured Interview") OR (MH "Interviews+") OR (MH "Focus Groups") OR "focus group*" OR (MH "Observational Methods+") OR "mixed-method*" OR (MH "Open-Ended Questionnaires") OR "open-ended*" OR (MH "Grounded Theory") OR "grounded theory" OR "thematic analysis" OR (MH "Life Experiences+") OR "lived experience*" OR "phenomenolog*" OR (MH "Phenomenological Research") OR "phenomenograph*" OR "ethnog*" OR (MH "Ethnographic Research") OR "autoethnograph*" OR "ethnomethodolog*" OR "narrative" OR (MH "Content Analysis") OR "content analysis" OR (MH "Discourse Analysis") OR "discourse analysis" OR "conversation analysis" OR (MH "Conceptual Framework") OR "reflexivity" OR "audiorecord*" OR "audio-record*" OR "videorecord*" OR (MH "Videorecording+") OR "video-record*")

**Supplementary Table 5.** EMBASE search strategy

**Emtree terms in bold**

1 gabapentinoid*.mp. 1769

2 gabapentin.mp. or **Gabapentin/** 44286

3 pregabalin.mp. or **Pregabalin/** 22571

4 lyrica.mp. 1336

5 neurontin.mp. 2189

6 mirogabalin.mp. 253

7 1 or 2 or 3 or 4 or 5 or 6 56636

8 misus*.mp. or **Drug misuse/** 46922

9 abus*.mp. 343933

10 nonmedical*.mp. or **Prescription Drug Misuse/** 10852

11 non-medical*.mp. 15059

12 nontherapeutic*.mp. 1038

13 non-therapeutic*.mp. 1807

14 extramedical*.mp. 70

15 extra-medical*.mp. 204

16 nonprescribed.mp. 398

17 non-prescribed.mp. 1087

18 nonprescription*.mp. 2320

19 non-prescription*.mp. 19640

20 substance us*.mp. 85596

21 **“Drug Use”/** or drug us*.mp. 251953

22 illicit.mp. 39148

23 (harmful adj3 us*).mp. [mp=title, abstract, heading word, drug trade name, original title, device manufacturer, drug manufacturer, device trade name, keyword heading word, floating subheading word, candidate term word] 4722

24 (risky adj3 us*).mp. [mp=title, abstract, heading word, drug trade name, original title, device manufacturer, drug manufacturer, device trade name, keyword heading word, floating subheading word, candidate term word] 2952

25 (high-risk adj3 us*).mp. [mp=title, abstract, heading word, drug trade name, original title, device manufacturer, drug manufacturer, device trade name, keyword heading word, floating subheading word, candidate term word] 14611

26 (high risk adj3 us*).mp. [mp=title, abstract, heading word, drug trade name, original title, device manufacturer, drug manufacturer, device trade name, keyword heading word, floating subheading word, candidate term word] 14611

27 (hazardous adj3 us*).mp. [mp=title, abstract, heading word, drug trade name, original title, device manufacturer, drug manufacturer, device trade name, keyword heading word, floating subheading word, candidate term word] 3763

28 (unsanctioned adj3 us*).mp. [mp=title, abstract, heading word, drug trade name, original title, device manufacturer, drug manufacturer, device trade name, keyword heading word, floating subheading word, candidate term word] 41

29 aberrant.mp. 164048

30 nonadherent.mp. 7137

31 non-adherent.mp. 9888

32 nonadherence.mp. 11826

33 non-adherence.mp. 18088

34 non-compliant.mp. 6136

35 noncompliant.mp. 4571

36 non-compliance.mp. 13763

37 noncompliance.mp. 11421

38 divert*.mp. 87519

39 diversion.mp. 42683

40 street.mp. 26308

41 tamper*.mp. 3350

42 intoxicate*.mp. 12999

43 intoxication.mp. 295569

44 recreation*.mp. 68128

45 euphori*.mp. 9770

46 polydrug*.mp. 2811

47 **Illicit Drugs/** 20491

48 poly-drug*.mp. 683

49 **Drug dependence/** or dependen*.mp. 3190886

50 **Addiction/** or addict*.mp. or **Drug dependence /** 256758

51 withdraw*.mp. 515082

52 **withdrawal syndrome/** 34578

53 toleran*.mp. 600360

54 **Craving/** or crav*.mp. 21092

55 habitua*.mp. 59149

56 detox*.mp. 81583

57 rehab*.mp. 536831

58 **Drug Overdose/** or overdose*.mp. 63252

59 wean*.mp. 97764

60 taper*.mp. 49155

61 discontinu*.mp. 302782

62 cessat*.mp. 169932

63 8 or 9 or 10 or 11 or 12 or 13 or 14 or 15 or 16 or 17 or 18 or 19 or 20 or 21 or 22 or 23 or 24 or 25 or 26 or 27 or 28 or 29 or 30 or 31 or 32 or 33 or 34 or 35 or 36 or 37 or 38 or 39 or 40 or 41 or 42 or 43 or 44 or 45 or 46 or 47 or 48 or 49 or 50 or 51 or 52 or 53 or 54 or 55 or 56 or 57 or 58 or 59 or 60 or 61 or 62 6409156

64 **Qualitative Research/** or qualitative.mp. 539561

65 interview*.mp. or **Interview/** or **semi structured interview/** 723628

66 Focus Groups/ or focus group*.mp. 357505

67 grounded theory.mp. or **Grounded Theory/** 21539

68 thematic analysis.mp. 75647

69 mixed-method*.mp. 64052

70 **questionnaire/** 1042795

71 open-ended.mp. 31979

72 lived experience*.mp. 19693

73 phenomenology.mp. 24676

74 phenomenological.mp. 30087

75 phenomenography.mp. 374

76 phenomenographical.mp. 44

77 ethnography.mp. 8181

78 ethnographical.mp. 173

79 ethnology.mp. or **Ethnology/** 84383

80 ethnological.mp. 365

81 ethnomethodology.mp. 133

82 ethnomethodological.mp. 101

83 autoethnography.mp. 333

84 autoethnographical.mp. 14

85 narrativ*.mp. 113294

86 Personal Narrative/ 61733

87 content analysis.mp. 63977

88 discourse analysis.mp. 4087

89 conversation analysis.mp. 1238

90 theme*.mp. 212785

91 thematic*.mp. 118571

92 reflexiv*.mp. 13649

93 audiorecord*.mp. 984

94 audio-record*.mp. 21603

95 videorecord*.mp. 137046

96 video-record*.mp. 17740

97 videotap*.mp. 16575

98 video-tap*.mp. 1910

99 taperecord*.mp. 72

100 tape-record*.mp. 5472

101 64 or 65 or 66 or 67 or 68 or 69 or 70 or 71 or 72 or 73 or 74 or 75 or 76 or 77 or 78 or 79 or 80 or 81 or 82 or 83 or 84 or 85 or 86 or 87 or 88 or 89 or 90 or 91 or 92 or 93 or 94 or 95 or 96 or 97 or 98 or 99 or 100 2713258

102 7 and 63 and 101 1454

**Supplementary Table 6.** PsycINFO search strategy

**APA Thesaurus of Psychological Index Terms in bold**

1 gabapentinoid*.mp. 167

2 gabapentin.mp. or exp **Gabapentin/** 1722

3 pregabalin.mp. or exp **Pregabalin/** 1184

4 lyrica.mp. 26

5 neurontin.mp. 33

6 mirogabalin.mp. 12

7 1 or 2 or 3 or 4 or 5 or 6 2642

8 misus*.mp. 18782

9 abus*.mp. 217648

10 nonmedical*.mp. or exp **Prescription Drug Misuse/** 3673

11 non-medical*.mp. 2929

12 nontherapeutic*.mp. 383

13 non-therapeutic*.mp. 309

14 extramedical*.mp. 42

15 extra-medical*.mp. 67

16 nonprescribed.mp. 162

17 non-prescribed.mp. 303

18 nonprescription*.mp. 1203

19 non-prescription*.mp. 291

20 substance us*.mp. 84456

21 drug us*.mp. 66389

22 illicit.mp. 13729

23 (harmful adj3 us*).mp. [mp=title, abstract, heading word, table of contents, key concepts, original title, tests & measures, mesh word] 1662

24 (risky adj3 us*).mp. [mp=title, abstract, heading word, table of contents, key concepts, original title, tests & measures, mesh word] 2179

25 (high-risk adj3 us*).mp. [mp=title, abstract, heading word, table of contents, key concepts, original title, tests & measures, mesh word] 2233

26 (high risk adj3 us*).mp. [mp=title, abstract, heading word, table of contents, key concepts, original title, tests & measures, mesh word] 2233

27 (hazardous adj3 us*).mp. [mp=title, abstract, heading word, table of contents, key concepts, original title, tests & measures, mesh word] 1186

28 (unsanctioned adj3 us*).mp. [mp=title, abstract, heading word, table of contents, key concepts, original title, tests & measures, mesh word] 28

29 aberrant.mp. 11852

30 nonadherent.mp. 555

31 non-adherent.mp. 640

32 nonadherence.mp. 2434

33 non-adherence.mp. 2376

34 non-compliant.mp. 477

35 noncompliant.mp. 1122

36 non-compliance.mp. 1539

37 noncompliance.mp. 3624

38 divert*.mp. 3148

39 diversion.mp. 3748

40 street.mp. 15118

41 tamper*.mp. 455

42 intoxicate*.mp. 2727

43 intoxication.mp. 12783

44 recreation*.mp. 23595

45 euphori*.mp. 2794

46 polydrug*.mp. 2607

47 exp **Drug Dependency/** or exp **Drug Abuse/** or exp **Drug Addiction/** or exp **Addiction/** or exp **"Substance Use Disorder"/** or exp **Illegal Drug Distribution/** or exp **Drug Addiction/** 155697

48 poly-drug*.mp. 341

49 dependen*.mp. 262768

50 addict*.mp. 80302

51 withdraw*.mp. 51443

52 exp **Drug Withdrawal/** or exp **Drug Overdoses/** 13751

53 toleran*.mp. 38539

54 **Craving/** or crav*.mp. 12542

55 habitua*.mp. 24198

56 detox*.mp. 5548

57 rehab*.mp. 89911

58 overdose*.mp. 8578

59 wean*.mp. 5954

60 taper*.mp. 2450

61 discontinu*.mp. 25545

62 cessat*.mp. 30124

63 8 or 9 or 10 or 11 or 12 or 13 or 14 or 15 or 16 or 17 or 18 or 19 or 20 or 21 or 22 or 23 or 24 or 25 or 26 or 27 or 28 or 29 or 30 or 31 or 32 or 33 or 34 or 35 or 36 or 37 or 38 or 39 or 40 or 41 or 42 or 43 or 44 or 45 or 46 or 47 or 48 or 49 or 50 or 51 or 52 or 53 or 54 or 55 or 56 or 57 or 58 or 59 or 60 or 61 or 62 843362

64 exp **Qualitative Methods/** or exp **Qualitative Measures/** or qualitative.mp. 288312

65 interview*.mp. or exp **Semi-Structured Interview/** 536524

66 exp **Focus Group/** or focus group*.mp. 54436

67 exp **Grounded Theory/** or grounded theory.mp. 21716

68 thematic analysis.mp. or exp **Thematic Analysis/** 36140

69 mixed-method*.mp. 44917

70 exp **Surveys/** 16753

71 open-ended.mp. 26297

72 exp **Lived Experience/** or lived experience*.mp. 28369

73 phenomenology.mp. 31086

74 phenomenological.mp. 46266

75 phenomenography.mp. 371

76 phenomenographical.mp. 61

77 ethnography.mp. 18225

78 ethnographical.mp. 295

79 exp **Ethnology/** or Ethnology.mp. 2512

80 ethnological.mp. 391

81 ethnomethodology.mp. 695

82 ethnomethodological.mp. 468

83 autoethnography.mp. 1972

84 autoethnographical.mp. 70

85 narrativ*.mp. 98993

86 exp **Narratives/**  27891

87 content analysis.mp. 40332

88 discourse analysis.mp. 15910

89 conversation analysis.mp. 3184

90 theme*.mp. 171272

91 thematic*.mp. 64855

92 reflexiv*.mp. 15577

93 audiorecord*.mp. 248

94 audio-record*.mp. 7525

95 videorecord*.mp. 446

96 video-record*.mp. 11191

97 videotap*.mp. 24186

98 video-tap*.mp. 1269

99 taperecord*.mp. 63

100 tape-record*.mp. 5228

101 64 or 65 or 66 or 67 or 68 or 69 or 70 or 71 or 72 or 73 or 74 or 75 or 76 or 77 or 78 or 79 or 80 or 81 or 82 or 83 or 84 or 85 or 86 or 87 or 88 or 89 or 90 or 91 or 92 or 93 or 94 or 95 or 96 or 97 or 98 or 99 or 100 954007

102 7 and 63 and 101 113

**Supplementary Table 7.** Scopus search strategy

( TITLE-ABS-KEY ( gabapentinoid*  OR  gabapentin  OR  pregabalin  OR  lyrica  OR  neurontin  OR  mirogabalin ) )  AND  ( ( TITLE-ABS-KEY ( misus*  OR  abus*  OR  nonmedical*  OR  non-medical*  OR  nontherapeutic*  OR  non-therapeutic*  OR  extramedical*  OR  extra-medical*  OR  nonprescribed  OR  non-prescribed )  OR  TITLE-ABS-KEY ( nonprescription*  OR  non-prescription*  OR  "substance us*"  OR  "drug us*"  OR  illicit  W/3  us*  OR  harmful  W/3  us*  OR  risky  W/3  us*  OR  high-risk  W/3  us*  OR  hazardous  W/3  us*  OR  unsanctioned  W/3  us* )  OR  TITLE-ABS-KEY ( aberrant  OR  nonadheren*  OR  non-adheren*  OR  non-complian*  OR  noncomplian*  OR  diversion  OR  divert*  OR  street  OR  tamper*  OR  intoxicat*  OR  recreation*  OR  euphori*  OR  polydrug  OR  poly-drug )  OR  TITLE-ABS-KEY ( dependen*  OR  addict*  OR  withdraw*  OR  toleran*  OR  crav*  OR  habitua*  OR  rehab*  OR  overdos*  OR  wean*  OR  taper*  OR  discontinue*  OR  cessat* ) ) )  AND  ( ( TITLE-ABS-KEY ( qualitative  OR  interview*  OR  {focus group*}  OR  {mixed method*}  OR  mixed-method*  OR  open-ended  OR  {open ended}  OR  {grounded theory}  OR  {thematic analysis}  OR  "lived experience*" )  OR  TITLE-ABS-KEY ( phenomenolog*  OR  phenomenograph*  OR  ethnolog*  OR  ethnog*  OR  autoethnograph*  OR  ethnomethodolog*  OR  narrativ*  OR  {content analysis}  OR  {discourse analysis} )  OR  TITLE-ABS-KEY ( {conversation analysis}  OR  theme*  OR  reflexivity  OR  audiorecord*  OR  audio-record*  OR  videorecord*  OR  video-record*  OR  videotap*  OR  video-tap*  OR  taperecord*  OR  tape-record* ) ) )

**Supplementary Table 8.** Web of Science search strategy

TS=(gabapentinoid* OR gabapentin OR pregabalin OR lyrica OR neurontin OR mirogabalin)

AND

TS=(misus* OR abus* OR nonmedical* OR non-medical* OR nontherapeutic* OR non-therapeutic* OR extramedical* OR extra-medical* OR nonprescribed OR non-prescribed OR nonprescription* OR non-prescription* OR "substance use*" OR "drug use*" OR illicit NEAR/3 use* OR harmful NEAR/3 use* OR risky NEAR/3 use* OR high-risk NEAR/3 use* OR hazardous NEAR/3 use* OR unsanctioned NEAR/3 use* OR aberrant OR nonadheren* OR non-adheren* OR non-complian* OR noncomplian* OR diversion OR divert* OR street OR tamper* OR intoxicat* OR recreation* OR euphori* OR polydrug OR poly-drug OR dependen* OR addict* OR withdraw* OR toleran* OR crav* OR habitua* OR rehab* OR overdos* OR wean* OR taper* OR discontinue* OR cessat*)

AND

TS=(qualitative OR interview* OR "focus group*" OR "mixed method*" OR mixed-method* OR open-ended OR "open ended" OR "grounded theory" OR "thematic analysis" OR "lived experience*" OR phenomenolog* OR phenomenograph* OR ethnolog* OR ethnog* OR autoethnograph* OR ethnomethodolog* OR narrativ* OR "content analysis" OR "discourse analysis" OR "conversation analysis" OR theme* OR reflexivity OR audiorecord* OR audio-record* OR videorecord* OR video-record* OR videotap* OR video-tap* OR taperecord* OR tape-record*)

**Supplementary Table 9.** ProQuest search strategy

noft(gabapentinoid* OR gabapentin OR pregabalin OR lyrica OR neurontin OR mirogabalin) AND noft(misus* OR abus* OR nonmedical* OR non-medical* OR nontherapeutic* OR non-therapeutic* OR extramedical* OR extra-medical* OR nonprescribed OR non-prescribed OR nonprescription* OR non-prescription* OR "substance use*" OR "drug use*" OR illicit NEAR/3 use* OR harmful NEAR/3 use* OR risky NEAR/3 use* OR high-risk NEAR/3 use* OR hazardous NEAR/3 use* OR unsanctioned NEAR/3 use* OR aberrant OR nonadheren* OR non-adheren* OR non-complian* OR noncomplian* OR diversion OR divert* OR street OR tamper* OR intoxicat* OR recreation* OR euphori* OR polydrug OR poly-drug OR dependen* OR addict* OR withdraw* OR toleran* OR crav* OR habitua* OR rehab* OR overdos* OR wean* OR taper* OR discontinue* OR cessat*) AND noft(qualitative OR interview* OR "focus group*" OR "mixed method*" OR mixed-method* OR open-ended OR "open ended" OR "grounded theory" OR "thematic analysis" OR "lived experience*" OR phenomenolog* OR phenomenograph* OR ethnolog* OR ethnog* OR autoethnograph* OR ethnomethodolog* OR narrativ* OR "content analysis" OR "discourse analysis" OR "conversation analysis" OR theme* OR reflexivity OR audiorecord* OR audio-record* OR videorecord* OR video-record* OR videotap* OR video-tap* OR taperecord* OR tape-record*)

**Supplementary Table 10.** Google Scholar search strategy

("gabapentin" OR "pregabalin" OR "mirogabalin" OR "neurontin" OR "lyrica" OR "gabapentinoid") AND ("misuse" OR "abuse" OR "non-medical use" OR "substance use" OR "drug use" OR "addiction" OR "dependence" OR "withdrawal") AND ("qualitative" OR "interview" OR "focus group" OR "grounded theory" OR "thematic analysis" OR "lived experience" OR "narrative" OR "content analysis")

**Supplementary Table 11.** Consensus CASP appraisal

| Paper | CASP questions | | | | | | | | | | |  | | |
| --- | --- | --- | --- | --- | --- | --- | --- | --- | --- | --- | --- | --- | --- | --- |
|  | 1. Was there a clear statement of the aims of the research? | 2. Is a qualitative methodology appropriate? | 3. Was the research design appropriate to address the aims of the research? | 4. Are the study’s theoretical underpinnings clear, consistent, and conceptually coherent? | 5. Was the recruitment strategy appropriate to the aims of the research? | 6. Was the data collected in a way that addressed the research issue? | 7. Has the relationship between researcher and participants been adequately considered? | 8. Have ethical issues been taken into consideration? | 9. Was the data analysis sufficiently rigorous? | 10. Is there a clear statement of findings? | 11. How valuable is the research? | | Overall appraisal of quality |  |
| Altayeb et al., 2025 | Yes | Yes | Somewhat | Somewhat | Yes | Somewhat | No | Yes | No | Somewhat | Somewhat | | Low |  |
| Buttram et al., 2017 | Yes | Yes | Somewhat | Somewhat | Somewhat | Somewhat | No | Yes | No | Somewhat | Somewhat | | Low |  |
| Buttram et al., 2019 | Yes | Yes | Yes | Somewhat | Yes | Yes | Somewhat | Somewhat | Yes | Yes | Valuable | | High |  |
| Buttram et al., 2023 | Yes | Yes | Somewhat | Somewhat | Somewhat | Somewhat | No | Yes | Somewhat | Yes | Valuable | | Medium |  |
| Chandrasiri et al., 2024 | Yes | Yes | Yes | No | Somewhat | Yes | No | No | Somewhat | Somewhat | Valuable | | Low |  |
| Coombes & Cooper, 2019 | Yes | Yes | Yes | Somewhat | Yes | Yes | Somewhat | Yes | Yes | Yes | Valuable | | High |  |
| Covvey et al., 2022; Covvey et al., 2023 | Yes | Yes | Yes | Yes | Yes | Yes | Somewhat | Yes | Yes | Yes | Valuable | | High |  |
| De Kock et al., 2023 | Yes | Yes | Yes | Can’t tell | Yes | Yes | Can’t tell | Can’t tell | Can’t tell | Yes | Valuable | | Medium |  |
| Falzon et al., 2023 | Yes | Yes | Yes | Yes | Yes | Yes | Somewhat | Yes | Yes | Yes | Valuable | | High |  |
| Ghinea et al., 2015 | Yes | Yes | Somewhat | Somewhat | Somewhat | Somewhat | No | Can’t tell | Somewhat | Somewhat | Somewhat | | Low |  |
| Gittins et al., 2024; Gittins, 2024 | Yes | Yes | Yes | Yes | Yes | Yes | Yes | Yes | Yes | Yes | Valuable | | High |  |
| Jeffries et al., 2025 | Yes | Yes | Yes | Yes | Yes | Yes | No | Yes | Yes | Yes | Valuable | | High |  |
| Jorgenson et al., 2021 | Yes | Yes | Yes | Somewhat | Yes | Yes | No | Yes | Somewhat | Yes | Somewhat | | Medium |  |
| Lehnus et al., 2024 | Yes | Yes | Somewhat | Yes | Somewhat | Yes | Somewhat | Yes | Somewhat | Yes | Valuable | | Medium |  |
| May et al., 2022 | Yes | Yes | Yes | Somewhat | Yes | Yes | Somewhat | Yes | Yes | Yes | Valuable | | High |  |
| Mikhael et al., 2024 | Yes | Yes | Yes | Somewhat | Yes | Yes | Somewhat | Yes | Yes | Yes | Valuable | | High |  |
| Mohamed et al., 2024 | Yes | Yes | Yes | No | Somewhat | Somewhat | No | Somewhat | No | Somewhat | Somewhat | | Low |  |
| Parbery-Clark et al., 2025 | Yes | Yes | Yes | Yes | Yes | Yes | Yes | Yes | Yes | Yes | Valuable | | High |  |
| Pivovarova et al., 2023 | Yes | Yes | Yes | Yes | Yes | Yes | Somewhat | Yes | Yes | Yes | Valuable | | High |  |

**Supplementary Table 12.** GRADE-CERQual Evidence Profile

| **#** | **Summarised review finding** | **Methodological limitations** | **Coherence** | **Adequacy** | **Relevance** | **GRADE-CERQual assessment of confidence** | **References** |
| --- | --- | --- | --- | --- | --- | --- | --- |
| 1 | *Variability in awareness:* Awareness of gabapentinoid misuse and dependence varied across countries, professional roles, and settings. While some professionals gained awareness through education or media, most cited first-hand exposure. Healthcare practitioners in psychiatry, community pharmacy, and substance use treatment were generally more alert to misuse than those in primary care. Misuse was also observed among vulnerable groups, including schoolchildren and asylum seekers. | Minor concerns  **Explanation:** 10 studies with no/very minor concerns, four studies with minor concerns, five studies with serious concerns. Data from studies with serious concerns – primarily related to research design and analytical rigour – were used only for contextual support. | Minor concerns  **Explanation:** In some cases, awareness appeared to be implied rather than explicitly stated by participants, and the synthesis may overgeneralise distinctions between medical settings, particularly between primary care and specialised services. | No/Very minor concerns | Minor concerns  **Explanation:** Six studies with direct relevance, eight studies with partial relevance, five studies with indirect relevance. Studies span diverse professional roles and countries, albeit predominantly conducted in North America and Europe. While many studies focused on substance use more generally, they provided meaningful insights relevant to this finding. | Moderate confidence  **Explanation:** Minor concerns regarding methodological limitations, Minor concerns regarding coherence, No/Very minor concerns regarding adequacy, and Minor concerns regarding relevance. | Altayeb et al. 2025; Buttram et al. 2017; Buttram et al. 2019; Buttram et al. 2023; Chandrasiri et al. 2024; Coombes & Cooper 2019; Covvey et al. 2023; De Kock et al. 2023; Falzon et al. 2023; Ghinea et al. 2015; Gittins 2024; Jeffries et al. 2025; Jorgenson et al. 2021; Lehnus et al. 2024; May et al. 2022; Mikhael et al. 2024; Mohamed & Bashir 2024; Parbery-Clark et al. 2025; Pivovarova et al. 2023; |
| 2 | *Divergent risk perceptions:* Healthcare practitioners generally perceived gabapentinoids as lower risk than opioids, often viewing them as a safer alternative in pain management. Law enforcement officers and professionals in disadvantaged or high-risk settings expressed more concern about potential harms associated with gabapentinoids, particularly when used in combination with opioids. | Minor concerns  **Explanation:** Six studies with no/very minor concerns, one study with minor concerns, two studies with serious concerns. Data from studies with serious concerns – primarily related to research design and analytical rigour – were used only for contextual support. | Minor concerns  **Explanation:** There may be some overgeneralisation in describing healthcare practitioner perceptions as low-risk. | Minor concerns  **Explanation:** Nine studies that together contributed moderately rich data. | Moderate concerns  **Explanation:** Four studies with direct relevance, two studies with partial relevance, three studies with indirect relevance. Studies span diverse healthcare roles and settings. However, all studies were conducted in the UK or US, limiting transferability. | Moderate confidence  **Explanation:** Minor concerns regarding methodological limitations, No/Very minor concerns regarding coherence, Minor concerns regarding adequacy, and Moderate concerns regarding relevance | Buttram et al. 2017; Buttram et al. 2019; Covvey et al. 2023; Falzon et al. 2023; Ghinea et al. 2015; Jeffries et al. 2025; Lehnus et al. 2024; Parbery-Clark et al. 2025; Pivovarova et al. 2023 |
| 3 | *Perceived drivers:* Gabapentinoid misuse was attributed to multiple drivers, including the pursuit of euphoria, enhancement of opioid effects, self-medication, drug availability, and undetected use in drug screens. Systemic issues such as poor access to non-pharmacological care, opioid tapering practices, and socioeconomic disadvantage were also implicated as key contributors. | Minor concerns  **Explanation:** Seven studies with no/very minor concerns, three studies with minor concerns, three studies with serious concerns. Data from studies with serious concerns – primarily related to research design and analytical rigour – were used only for contextual support. | No/Very minor concerns | No/Very minor concerns | Minor concerns  **Explanation:** Five studies with direct relevance, seven studies with partial relevance, one study with indirect relevance. Studies span diverse professional roles, settings, and countries. | High confidence  **Explanation:** Minor concerns regarding methodological limitations, No/Very minor concerns regarding coherence, No/Very minor concerns regarding adequacy, and Minor concerns regarding relevance | Altayeb et al. 2025; Buttram et al. 2017; Buttram et al. 2019; Buttram et al. 2023; Coombes & Cooper 2019; Covvey et al. 2023; De Kock et al. 2023; Gittins 2024; Lehnus et al. 2024; May et al. 2022; Mikhael et al. 2024; Mohamed & Bashir 2024; Parbery-Clark et al. 2025 |
| 4 | *Potential signs:* Participants identified behavioural and prescribing patterns as signs of misuse or dependence, including early refills, unsanctioned dose escalation, multiple prescribers, and signs of intoxication or withdrawal. Due to stigma and legal concerns, patients often concealed misuse, prompting practitioners to rely on indirect indicators*.* | Minor concerns  **Explanation:** Four studies with no/very minor concerns, two studies with minor concerns, one study with serious concerns. Data from studies with serious concerns – primarily related to research design and analytical rigour – were used only for contextual support. | Minor concerns  **Explanation:** Some studies provided examples of misuse signs without clearly attributing them to gabapentinoids, making it uncertain whether the finding fully reflects only gabapentinoid-specific patterns. | Moderate concerns  **Explanation:** Seven studies with several contributing relatively thin data. | Minor concerns  **Explanation:** Three studies with direct relevance, three studies with partial relevance, one study with indirect relevance. Studies span diverse professional roles and countries. Predominantly conducted in North America and the UK. | Moderate confidence  **Explanation:** Minor concerns regarding methodological limitations, Minor concerns regarding coherence, Moderate concerns regarding adequacy, and Minor concerns regarding relevance | Buttram et al. 2017; Buttram et al. 2019; Covvey et al. 2023; Jorgenson et al. 2021; Lehnus et al. 2024; Mikhael et al. 2024; Parbery-Clark et al. 2025 |
| 5 | *Navigating ethical dilemmas:* Professionals described complex ethical tensions in managing gabapentinoid prescribing and dispensing, particularly in the context of chronic pain, substance use, and social disadvantage. They often faced a difficult balance between alleviating patient suffering and avoiding the potential for misuse or dependence. Decision-making was further complicated by limited clinical guidance, poor access to alternatives, concerns about damaging therapeutic relationships, and, in some cases, patient threats of self-harm. | No/Very minor concerns  **Explanation:** Four studies with no/very minor concerns, one study with serious concerns. Serious concerns related to weakness in research design and lack of analytical rigour. Study with serious concerns contributed very limited data. | Minor concerns  **Explanation: Data** from one study referred to both gabapentinoids and opioids, making it unclear at times whether ethical dilemmas related specifically to gabapentinoids. | No/Very minor concerns | No/Very minor concerns | High confidence  **Explanation:** No/Very minor concerns regarding methodological limitations, Minor concerns regarding coherence, No/Very minor concerns regarding adequacy, and No/Very minor concerns regarding relevance | Buttram et al. 2019; Covvey et al. 2023; Ghinea et al. 2015; Mikhael et al. 2024; Parbery-Clark et al. 2025 |
| 6 | *Improving clinical decision-making:* Strategies to support safer prescribing included improved education, clearer guidelines, better monitoring systems (e.g., PDMPs, decision support tools), and enhanced interdisciplinary collaboration. Early prescribing decisions were seen as crucial, particularly for high-risk patients, and professionals called for more robust tools and training to manage tapering and dependence. | No/Very minor concerns  **Explanation:** Seven studies with no/very minor concerns, one study with minor concerns, one study with serious concerns. Serious concerns related to weakness in research design and lack of analytical rigour. Study with serious concerns contributed very limited data. | Minor concerns  **Explanation:** Some supporting data were not clearly specific to gabapentinoids. | Minor concerns  **Explanation:** Nine studies that together contributed moderately rich data. | No/Very minor concerns | Moderate confidence  **Explanation:** No/Very minor concerns regarding methodological limitations, Minor concerns regarding coherence, Minor concerns regarding adequacy, and No/Very minor concerns regarding relevance | Altayeb et al. 2025; Buttram et al. 2019; Coombes & Cooper 2019; Covvey et al. 2023; Gittins 2024; Jeffries et al. 2025; Jorgenson et al. 2021; Mikhael et al. 2024; Parbery-Clark et al. 2025 |
| 7 | *Strengthening regulation and enforcement:* Regulatory and enforcement responses were seen as important but potentially double-edged. While scheduling gabapentinoids was supported by law enforcement, healthcare practitioners and drug policy experts expressed concern about patient access and unintended consequences. Participants stressed the need for balanced regulation that protects public health without being overly punitive. | Moderate concerns  **Explanation:** Two studies with no/very minor concerns, two studies with minor concerns, two studies with serious concerns. Serious concerns related to weakness in research design and lack of analytical rigour. Although studies with serious concerns were deprioritised during theme development, their data featured prominently in the reporting of the finding. | Minor concerns  **Explanation:** Some supporting data were not clearly specific to gabapentinoids. | Moderate concerns  **Explanation:** Six studies with several contributing relatively thin data. | Minor concerns  **Explanation:** Three studies with direct relevance, two studies with partial relevance, one study with indirect relevance. Majority of data from two US studies and views on scheduling highly specific to that context, limiting transferability. | Low confidence  **Explanation:** Moderate concerns regarding methodological limitations, Minor concerns regarding coherence, Moderate concerns regarding adequacy, and Minor concerns regarding relevance | Altayeb et al. 2025; Buttram et al. 2023; Covvey et al. 2023; Jorgenson et al. 2021; Mikhael et al. 2024; Mohamed & Bashir 2024 |
| 8 | *Expanding access to care:* Improving access to non-pharmacological pain care, substance use treatment, and mental health support was seen as vital for prevention and harm reduction. Barriers included cost, availability, stigma, and systemic underinvestment. Participants emphasised the need for holistic, inclusive services tailored to the needs of people with chronic pain or prescription drug dependence. | Minor concerns  **Explanation:** Four studies with no/very minor concerns, two studies with serious concerns. Data from studies with serious concerns – primarily related to research design and analytical rigour – were used only for contextual support. | Moderate concerns  **Explanation:** Some supporting data were not clearly specific to gabapentinoids, and conclusion may suggest varied participant views were more cohesive or consistent than the primary data suggest. | Minor concerns  **Explanation:** Six studies that together contributed moderately rich data. | No/Very minor concerns | Moderate confidence  **Explanation:** Minor concerns regarding methodological limitations, Minor concerns regarding coherence, Minor concerns regarding adequacy, and No/Very minor concerns regarding relevance | Altayeb et al. 2025; Coombes & Cooper 2019; Covvey et al. 2023; Gittins 2024; Mohamed & Bashir 2024; Parbery-Clark et al. 2025 |
| 9 | *Raising awareness:* Participants highlighted the importance of coordinated awareness initiatives to improve patient understanding and reduce stigma. Strategies included patient education at the point of prescribing, public health campaigns, and community engagement. However, limited resources, unclear leadership, and clinical constraints hindered widespread implementation. | Minor concerns  **Explanation:** Four studies with no/very minor concerns, two studies with serious concerns. Data from studies with serious concerns – primarily related to research design and analytical rigour – were used only for contextual support. | Moderate concerns  **Explanation:** Much of the supporting data were not clearly specific to gabapentinoids, and the conclusion may overgeneralise diverse and context-specific participant perspectives into a unified call for coordinated awareness efforts. | Minor concerns  **Explanation:** Six studies that together contributed moderately rich data. | Moderate concerns  **Explanation:** One study with direct relevance, four studies with partial relevance, one study with indirect relevance. Four studies focused on substances use generally with gabapentinoids only briefly mentioned. | Low confidence  **Explanation:** Minor concerns regarding methodological limitations, Moderate concerns regarding coherence, Minor concerns regarding adequacy, and Moderate concerns regarding relevance | Altayeb et al. 2025; Coombes & Cooper 2019; Falzon et al. 2023; Gittins 2024; Mohamed & Bashir 2024; Parbery-Clark et al. 2025 |
